# Deleterious and ethnic-related *BRCA1/2* mutations in tissue and blood of Egyptian colorectal cancer patients and its correlation with human papillomavirus

**DOI:** 10.1007/s10238-023-01207-w

**Published:** 2023-10-07

**Authors:** Amira Salah El-Din Youssef, Abdel Rahman N. Zekri, Marwa Mohanad, Samah A. Loutfy, Nasra F. Abdel Fattah, Mostafa H. Elberry, Asmaa A. El Leithy, Ahmed El-Touny, Ahmed Samy Rabie, Mohamed Shalaby, Ayman Hanafy, Mai M. Lotfy, Enas R. El-sisi, Gharieb S. El-Sayyad, Auhood Nassar

**Affiliations:** 1 Virology and Immunology Unit, Cancer Biology Department, National Cancer Institute, Cairo University, Cairo, Egypt; 2 Department of Biochemistry, College of Pharmaceutical Sciences and Drug Manufacturing, Misr University for Science and Technology, Giza, Egypt; 3 Department of Medical Biotechnology, College of Biotechnology, Misr University for Science and Technology, Giza, Egypt; 4 Surgical Oncology Department, National Cancer Institute, Cairo University, Cairo, Egypt; 5 Department of Microbiology and Immunology, Faculty of Pharmacy, Ahram Canadian University, Giza, Egypt; 6 Department of Microbiology and Immunology, Faculty of Pharmacy, Galala University, New Galala City, Suez, Egypt; 7 Drug Microbiology Lab., Drug Radiation Research Department, National Center for Radiation Research and Technology (NCRRT), Egyptian Atomic Energy Authority (EAEA), Cairo, Egypt; 8 Nanotechnology Research Center (NTRC), The British University in Egypt (BUE), El-Shorouk City, Suez Desert Road, P. O. Box 43, Cairo, Egypt

**Keywords:** Egyptian colorectal cancer, *BRCA1*, *BRCA2*, Pathogenic, Next-generation, Sequencing

## Abstract

This study aimed to identify *BRCA1/2* mutational patterns in the tissue and blood of Egyptian colorectal cancer (CRC) patients and to study the possible correlation of this mutational pattern with Human papillomavirus (HPV) infection. Eighty-two colonoscopic biopsies and forty-six blood samples were collected from Egyptian CRC patients, as well as blood samples of age and sex-matched healthy controls (n = 43) were enrolled. The libraries were performed using Qiaseq Human *BRCA1* and *BRCA2* targeted DNA panel and sequenced via Ion proton sequencer. Also, the CRC tissues were subjected to conventional PCR targeting the HPV Late 1 (L1) region. Our analysis revealed that the *BRCA*-DNA damage pathway had been altered in more than 65% of the CRC patients. Comparing tissue and blood samples from CRC patients, 25 somatic mutations were found exclusively in tissue, while 41 germline mutations were found exclusively in blood. Additionally, we identified 23 shared *BRCA1/2* pathogenic (PVs) mutations in both blood and tissue samples, with a significantly higher frequency in blood samples compared to tissue samples. The most affected exon in *BRCA1* was exon 10, while the most affected exons in *BRCA2* were 11, 14, 18, 24, and 27 exons. Notably, we revealed an ethnic-related cluster of polymorphism variants in our population closely related to South Asian and African ethnicities. Novel PVs were identified and submitted to the ClinVar database. HPV was found in 23.8% of the CRC tissues, and 54% of HPV-positive cases had somatic *BRCA1/2* PVs. The results of this research point to a possible connection between infection with HPV and *BRCA1/2* mutations in the occurrence of colorectal cancer in the Egyptian population, which has a mixed ethnic background. Our data also indicate that liquid biopsy (blood samples) may be more representative than tissue samples for detecting *BRCA1/2* mutations. These findings may have implications for cancer screening and the development of personalized, targeted therapies, such as PARP inhibitors, which can effectively target *BRCA1/2* mutations.

**Supplementary Information:**

The online version contains supplementary material available at 10.1007/s10238-023-01207-w.

## Background

Colorectal cancer (CRC) is the second most prevalent cancer globally in both genders, ranking the third among diseases overall. It holds the unfortunate distinction of being the second leading cause of mortality attributed to cancer [1]. CRC was recognized as the sixth form of cancer among Egypt's most prevalent malignancies in 2013, based on the country's national cancer registry. Furthermore, it has been estimated that between 2013 and 2050, there would probably be an increase in the CRC cases [2].

CRC is characterized by complex molecular alterations. At least 10% of all CRC cases are attributed to germline genomic alterations. Human disease-causing variants are either germline and/or somatic variants. Critical key germline and somatic variants causing CRC worldwide were reported previously [3].

Beyond genetics, the global cancer landscape's intricacies reveal a link to infections. Recent data emphasize infection's role in altering global cancer incidence [4]. Furthermore, numerous DNA damage inductions were demonstrated during viral infection [5]. Numerous publications have also noted the connection between the risk of CRC and viral infections [6], particularly the human papillomavirus (HPV) [7, 8].

The identification of germline predispositions strategically holds promise for cancer management and prevention. Currently, DNA damage response (DDR) alterations are considered new therapeutic targets for different cancer types. About 10–20% of somatic DDR mutations were reported by previous studies in CRC [9, 10]. Additionally, the observed prevalence of shared somatic and germline genetic variations between blood and tissue samples may be attributed to the presence of circulating tumor cells (CTCs). These CTCs, originating from the primary tumor, have the potential to enter the bloodstream and subsequently seed new tumors in distant organs. While many of these genetic variants have been identified, their specific contribution to the risk of colorectal cancer (CRC) remains unclear [11].

The *BRCA1* and *BRCA2* genes are prominent in the DDR realm [12]. The increased risks of breast and ovarian cancer are linked to pathogenic variations (PVs) in *BRCA1* and *BRCA2* [13]. In addition, *BRCA1* and *BRCA2* PVs were related to risks for GIT cancers, including CRC, liver, and stomach [14].

According to recent recommendations, people with *BRCA1/2* PVs should consider participating in experimental screening trials and learning about the signs and symptoms of malignancies that may be related to their condition [15].

*BRCA* and other DDR complex genes are considered drug targets and treatment regimens in the majority of CRC patients [16]. Despite blocking molecular pathways by targeted drugs that have been used as adjunct to chemotherapy, CRC patients have not yet reaped significant benefits. Therefore, advanced research investigations are required to improve patient outcomes [17]. For patients with breast, ovarian, and most recently pancreatic cancer, targeted therapy using poly (ADP-ribose) polymerases inhibitors (PARPis) of *BRCA* mutation had improved patient survival [18]. This leads to the question of whether other cancers like CRC malignancies could benefit from targeted PARP inhibitors.

Next-generation sequencing (NGS) has made major advances in tumorigenesis, therapeutic target, and diagnostic markers of CRC [19]. Thus, it is worth mentioning that our study is the first to assess the comprehensive mutational profile of *BRCA1/2* in tissue and blood of CRC patients using NGS, as well as its correlation with HPV infection in Egypt.

## Material and methods

### Samples from patient

The National Cancer Institute (NCI) of Egypt provided colonoscopic biopsy samples (n = 82) from CRC patients. The obtained biopsies were preserved at − 80 °C in MACS Tissue Storage Solution till DNA extraction. In addition, blood samples were collected from CRC patients (n = 46) and healthy controls (n = 43) who were matched for age and gender.

The participant's clinicopathological information was gathered from their medical records at the National Cancer Institute (NCI). The Institutional Review Board of NCI, Cairo University, Egypt, authorized all protocols and procedures (IRB number: IRB00004025; approval number: 201617011.3). Each participant gave their written informed permission before being included in this study.

### DNA extraction

First, the DNA was extracted from the obtained biopsies using the QIAamp® DNA mini kit (Cat. No. 51304, Qiagen, Germany) following the manufacturer’s guidelines. The DNA was also extracted from whole blood samples using the QIAamp DNA Blood Mini Kit (Cat. No. 51104, Qiagen, Germany). Using the Qubit® 3.0 Fluorometer (Cat. No. Q33216, Thermo Fischer Scientific Inc., USA) and the QubitTM dsDNA HS assay kit (Cat. No. Q32854, Thermo Fischer Scientific Inc., USA), the concentration of the pure DNA was determined.

### HPV conventional PCR

The purified DNA (100 ng) extracted from the fresh tissue was subjected to the polymerase chain reaction (PCR) targeting the HPV Late 1 (L1) region. The Veriti 96-well quick thermal cycler (Cat. No. 4375305, Thermo Fischer Scientific Inc., USA) was used to perform the amplification. AmpliTaq Gold 360 PCR master mix (Cat. No. 4398881, Thermo Fischer Scientific Inc., USA) was used in the PCR reaction. Each PCR assay included positive and negative controls; the positive control for HPV was MCF7 (Michigan Cancer Foundation-7) cells. The primers, positive control, and cycling conditions were performed according to a previously published protocol by Metwally et al. [20].

### Library preparation and sequencing

We employed the QIAseq Human *BRCA1,* and *BRCA2* targeted DNA panel (Qiagen, Germany, Cat. No. DHS-102Z). The manufacturer's instructions were followed while building the NGS libraries. Then, the fragment size and concentration were assessed using the QIAxcel DNA high-resolution kit (Cat No. 929002, Qiagen, Hilden, NRW, Germany). The libraries were subsequently quantified using the QIAseq Library Quant Assay Kit (Cat No. 333304, available from Qiagen, Hilden, NRW, Germany). The template was prepared using the Ion PI Hi-Q Chef Kit (Cat. No. A27198, Thermo Fischer Scientific Inc., USA), and sequencing on the Ion Proton Platform was done using the Ion Proton Sequencing 200 Kit v2 (Cat. No. 4485149, Thermo Fischer Scientific Inc., USA).

### Bioinformatics analysis

Signal processing and base calling were performed using the Ion Torrent Suite. Variant calling and alignment were accomplished using the QIAGEN GeneGlobe Data Analysis Centre, in conjunction with the Annovar software, which incorporates population datasets. The read examination processes start with read processing steps that (i) remove exogenous sequences like PCR and sequencing adapters and UMI (unique molecular index), (ii) determine the UMI sequence and add it to the examine identifier for downstream evaluations, and (iii) eliminate short sequences that do not have enough endogenous sequence for mapping to the reference genome (hg19/GRCH37). Following trimming, reads are mapped to the reference genome, and reads that were poorly mapped (> Q30) are then filtered out. The aligned readings (in BAM format) are then forwarded on to variant calling using Sumcounter2 filters following UMI clustering. Only runs with depths greater than 100 and coverage of more than 95% of the target locations were considered successful. Synonymous and low-quality variants were filtered out as the filters are designed to catch false positive calls that have incorrectly high mutation likelihood for various reasons. A non-reference allele must pass the quality score threshold, and all filters to be reported as a variant.

To assess the germline variants detected in the CRC whole blood, the variants were compared to those found in the healthy controls to filter out normally inherited polymorphism, and the ethnic-related variants, as well as the founder pathogenic mutations found in the Egyptian population. The ethnic-related variants with high frequency, more than 30% in our population, were compared to the other population in the Exome Aggregation Consortium (ExAC) database. ExAC has recently been extended to the genomes (gnomAD) database which contains data from 141,456 individuals. This allows for an illustrative overview of the population and ethnic groups [21].The variants were considered shared if they were found in both tissue and blood of the CRC patients. We remove the variations discovered in the CRC blood (shared variants) and only keep the remaining variants in the tissue with an allele frequency of less than 0.5 to further determine that the remaining variants in the CRC tissues are somatic.

Functional consequences of the identified variants were predicted using Sift [22], PolyPhen-2 [23], and CADD [24] tools. Variant information was obtained using the dbSNP database (http://www.ncbi. nlm.nih.gov/projects/SNP), Human Gene Mutation Database (HGMD), Exac All Database (genomeAD), the 1000 Genome project, COSMIC Database and ClinVar database (http://www.ncbi.nlm.nih.gov/clinvar/). Mutations were classified according to American College of Medical Genetics and Genomics (ACMG) recommendations [25] into benign, likely benign, variants of uncertain significance (VUS), likely-pathogenic variant (LPV), and pathogenic variant (PV). In this study, we considered the variant pathogenic (deleterious) if it was classified as PV or LPV.

The novel variants predicted to be deleterious were submitted to the ClinVar submission portal (Organization ID: 507536; Genomic Center, National Cancer Institute, Egypt); Clinvar Link: https://www.ncbi.nlm.nih.gov/clinvar/submitters/507536/. For pathway analysis, we used Ingenuity Variant Analysis (IVA; QIAGEN, Germany).

### Statistical analysis

R studio statistical software (version 3.7, R Foundation for Statistical Computing, Vienna, Austria) was used for all statistical analyses. The pwr package was used to adjust the test power. The variant positions for *BRCA1* and *BRCA2* were depicted by a lollipop plot. The proportion of the most prevalent *BRCA1* and *BRCA2* variants in the investigated samples was shown using the oncoplots. Fischer's exact test was utilized to examine differentially altered gene variants in blood and tissue samples from different cohorts and to conduct pairwise or group-wise comparisons to study the relationship between *BRCA1* and *BRCA2* gene variations and clinical features. A forest plot was used to display the odd ratios for *BRCA1*/*BRCA2* in different groups. In all two-tailed tests needing a *P-value* of 0.05 to demonstrate significance, multiple comparisons were adjusted for the false discovery rate (FDR).

## Results

### Clinicopathological features of the CRC patients

Herein, we outlined the clinicopathological characteristics of 82 CRC patients. According to Table S1, these characteristics included age, gender, tumor pathology classifications, and recurrence and metastasis status. The average age of CRC patients was 50.48 years, and the male-to-female ratio was 1.1, according to our findings. Notably, approximately 50% of malignant tumors were found in the rectum, while 35% were found in the colon. Adenocarcinoma emerged as the predominant pathological subtype among CRC patients, accounting for 84% of cases. Regarding the degree of tumor differentiation, grade II and grade III were found in 60% and 23% of our CRC patients, respectively. In addition, the majority of CRC patients in our cohort had neither a cancer history nor a metastatic or recurrent condition.

### *BRCA1/2* mutational profile in the tissue of the CRC patients

Our pathway analysis using Ingenuity Variant Analysis (IVA) revealed that the inferred activity of *BRCA1*/2 mutations was loss of function and that the *BRCA*-DNA damage pathway has been altered significantly in more than 65% of the CRC patients compared to the healthy controls (*P-value* = 4.25E-16).

Upon analyzing the tissue of CRC patients and excluding synonymous mutations, a significant finding emerges. Specifically, the *BRCA2* gene mutations (total = 75) are more prevalent than the *BRCA1* gene mutations (total = 33). All *BRCA1* gene mutations are located on the negative DNA strand, whereas all *BRCA2* gene mutations are located on the positive DNA strand.

Intriguingly, the maximum number of mutations per patient in the *BRCA1* gene is eight, while the maximum number of mutations per patient in the *BRCA2* gene is 15. Additionally, these mutations exhibit distinct variant types. Seventy-three percent of the *BRCA1* variants are SNPs, followed by Deletion (Del) (21%) and Insertion (INS) (6%). In contrast, the most common class of variant for *BRCA2* is Del (47%), followed by SNP (41%) and INS (4%).

**Fig. 1:**
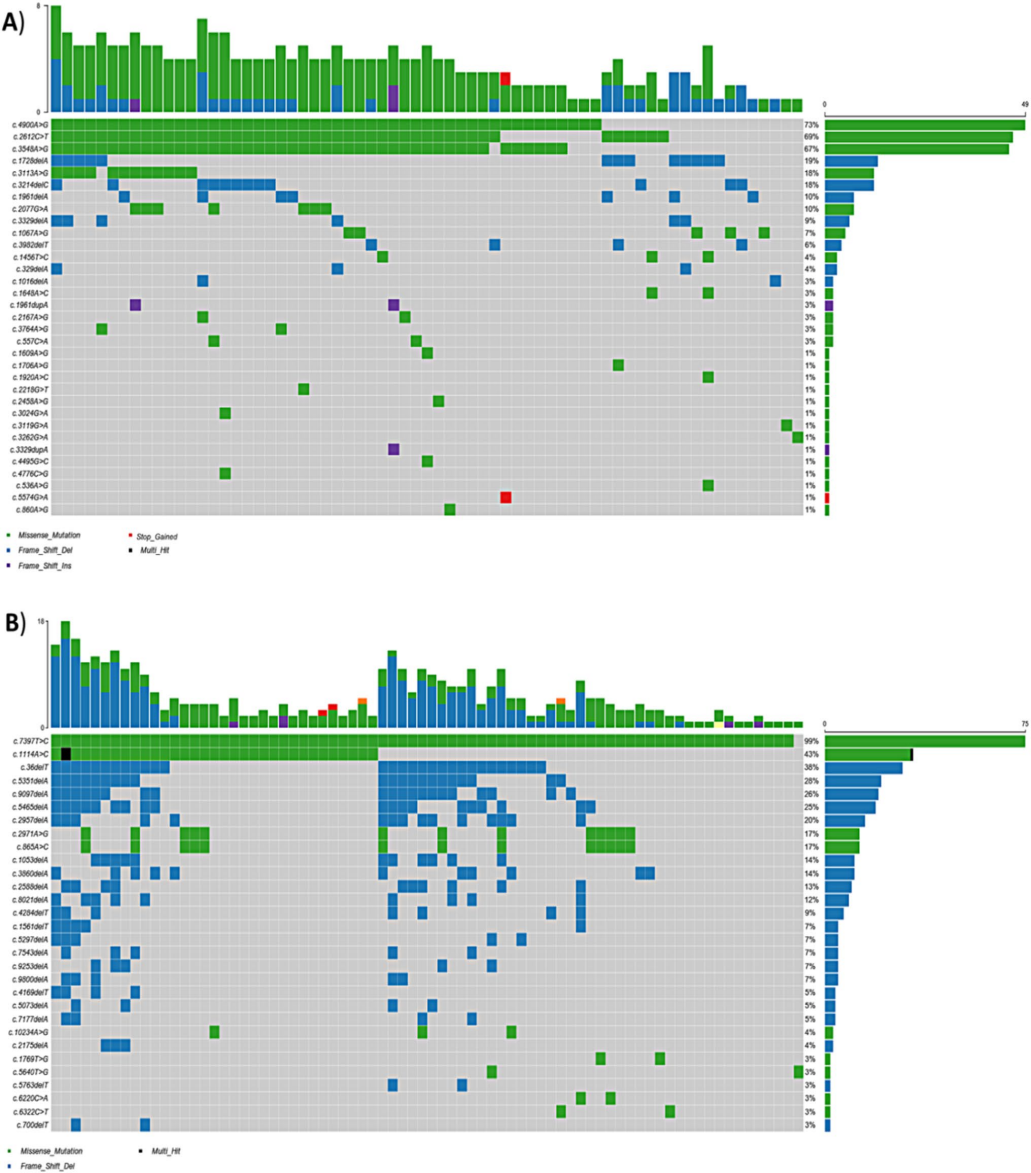
Oncoplots show the overall distribution of highly frequent **A** *BRCA1*, **B** *BRCA2* mutations in the tissue of the CRC patients. Each column represents a patient, and each row represents a variant. Different variants colors represent different classifications

When examining the specific SNP classes, a distinct pattern becomes apparent. The most common SNP type in the *BRCA1* gene entails the transition from the reference allele T to the alternative allele C, followed by the reversal. Likewise, the predominant SNP type in *BRCA2* is the transition from T to C, followed by the transversion from T to G (Fig. 1).

Figure S1 and Figure S2 provide a visual representation of the clinical significance of *BRCA1* and *BRCA2* gene variations in accordance with ACMG guidelines. These recommendations classify variants as benign, likely-benign, variants of uncertain significance (VUS), likely-pathogenic variant (LPV), and pathogenic variant (PV).

**Fig. 2:**
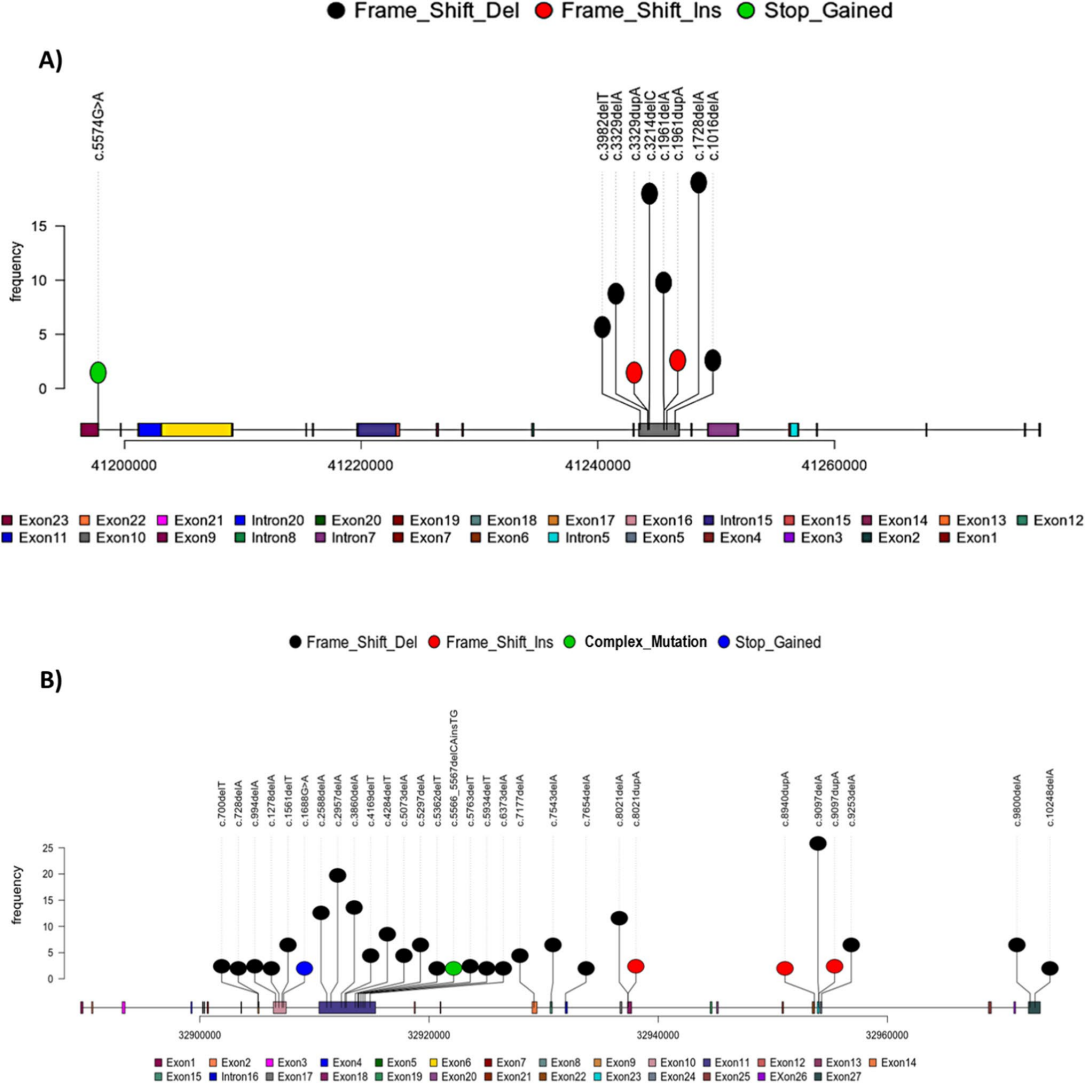
Lollipop representations show the location of the pathogenic mutations in **A** *BRCA1*, **B** *BRCA2* in the tissue of the CRC patients. The mutations are colored according to their type

**Fig. 3:**
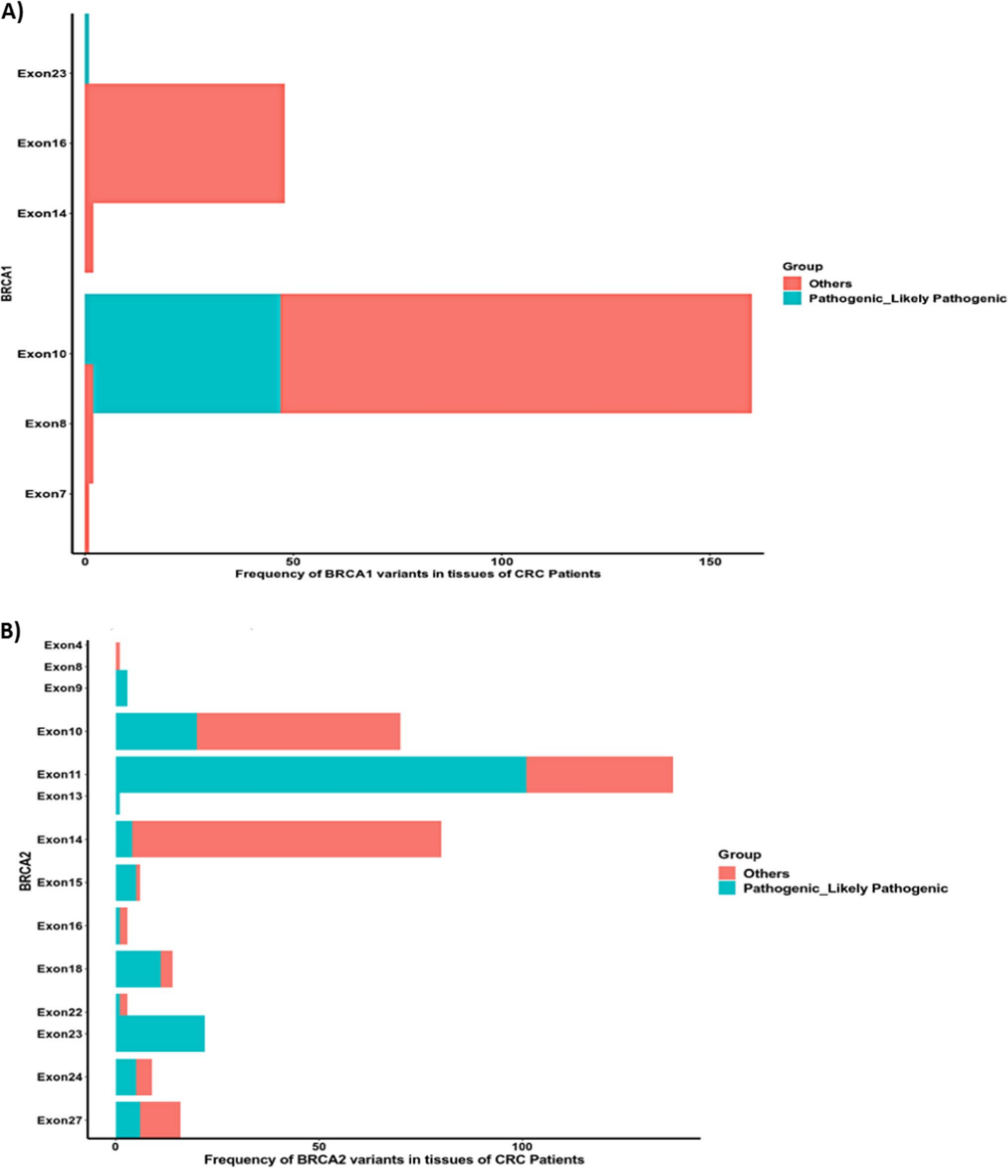
Bar graphs show the most affected exons harbored pathogenic and likely pathogenic mutations in **A** *BRCA1* and **B** *BRCA2* in the tissue of the CRC patients. The mutations are colored according to their type

The pathogenic variants (PVs) in *BRCA1* and *BRCA2* across different exons are illustrated in Figs. 2 and 3. It has been revealed that the most affected exons harboring PVs in *BRCA1* were exon 10 followed by exon 23, whereas the most affected exons harboring PVs in *BRCA2* were exon 11, followed by exon 10, exon 23, and exon 18.

Upon comparing the variants identified in the tissue to that in the blood of the CRC patients, we found nine and 16 somatic mutations in *BRCA1* and *BRCA2* genes*,* respectively. The somatic *BRCA1* mutations were classified according to their clinical significance into three PVs, four variants with uncertain significance (VUS), and two variants with conflicting interpretations of pathogenicity (CIP). The somatic *BRCA2* mutations were classified into eight VUS, three CIP, and five PVs. Notably, as indicated in Table 1, a novel complex variant (c.5566 5567delCAinsTG) was identified as a PV in exon13 *BRCA2* in one CRC patient.

**Table 1:** Somatic *BRCA1* and *BRCA2* mutations detected only in the tissues of Egyptian CRC patients

Gene	Position	Exon	ID	Type	Clinical Significance	HGVS.c	HGVS.p	CRC Tissue (n=82)	VMF	DP
*BRCA1*	Chr17:41245939	10	rs398122639, COSM9213250	SNP	CIP	c.1609A > G	p.Asn537Asp	1	0.13	738
	Chr17 41223155	10	rs761925468	SNP	CIP	c.4776C > G	p.Asn1592Lys	1	0.47	104
	Chr17: 41245586	10	rs80357853, COSM23947	INS	PV	c.1961dupA	p.Tyr655fs	2	0.22	340
	Chr17: 41244218	10	rs80357575	INS	PV	c.3329dupA	p.Gln1111fs	1	0.21	108
	Chr17: 41245842	10	rs1566224153, COSM3189989	SNP	VUS	c.1706A > G	p.Asn569Ser	1	0.18	110
	Chr17: 41245330	10	rs80357415, COSM7357696	SNP	VUS	c.2218G > T	p.Val740Leu	1	0.33	184
	Chr17: 41228557	10	COSM6943772, rs28897691	SNP	VUS	c.4495G > C	p.Glu1499Gln	1	0.04	1204
	Chr17: 41246688	10	rs1165149350	SNP	VUS	c.860A > G	p.Asn287Ser	1	0.30	330
	Chr17: 41197776	23	rs80356914, COSM10049588	SNP	PV	c.5574G > A	p.Trp1858*	1	0.14	103
*BRCA2*	Chr13: 32903577	7	rs568027879	SNP	CIP	c.632-3C > A	-	1	0.40	1408
	Chr13: 32903592	8	rs1198988757	SNP	VUS	c.644A > T	p.Glu215Val	1	0.18	290
	Chr13: 32907303	10	rs1566224110	SNP	PV	c.1688G > A	p.Trp563*	1	0.13	704
	Chr13: 32907384	10	rs80358459	SNP	CIP	c.1769T > G	p.Phe590Cys	2	0.49	110
	Chr13: 32906839	10	rs1593892275	SNP	VUS	c.1224G > C	p.Met408Ile	1	0.37	99
	Chr13: 32911602	11	rs1566227808	SNP	VUS	c.3110A > G	p.Gln1037Arg	1	0.12	978
	Chr13: 32911833	11	rs2053602865	SNP	VUS	c.3341T > C	p.Leu1114Pro	1	0.15	804
	Chr13: 32912344	11	rs777895333	SNP	VUS	c.3852T > A	p.Ser1284Arg	1	0.40	105
	Chr13: 32912873	11	rs1060502490	SNP	VUS	c.4381T > C	p.Ser1461Pro	1	0.23	576
	Chr13: 32913764	11	rs80358750	SNP	CIP	c.5272A > G	p.Asn1758Asp	1	0.11	628
	Chr13: 32914058	13	SCV001499586.1	Complex	NPV	c.5566_5567delCAinsTG	p.His1856Cys	1	0.08	216
	Chr13: 32930567	15	rs80358965	SNP	VUS	c.7438T > G	p.Leu2480Val	1	0.49	170
	Chr13: 32937354	18	COSM2071519, rs397507952	INS	PV	c.8021dupA	p.Ile2675fs	2	0.25	142
	Chr13: 32953632	22	COSM2071547, rs80359732	INS	PV	c.8940dupA	p.Glu2981fs	1	0.13	200
	Chr13: 32954022	23	COSM2071555, rs397507419	INS	PV	c.9097dupA	p.Thr3033fs	2	0.16	276
	Chr13: 32972489	27	rs80359246	SNP	VUS	c.9839C > A	p.Pro3280His	2	0.48	450

HGVS.c, Human Genome Variation Society, coding DNA sequence; HGVS.p, Human Genome Variation Society, protein sequence; Chr., Chromosome; CRC, Colorectal Cancer; PV, Pathogenic Variants; LPV, Likely Pathogenic Variant; VUS, Variants of Uncertain Significance; CIP, Conflicting Interpretation of Pathogenicity; NPV, Novel Pathogenic Variant; LNPV, Likely Novel Pathogenic variant; VMF, Variant Major Allele Frequency; SNP, Single Nucleotide Polymorphism; INS, Insertion; Del, Deletion; DP, Depth of coverage

### *BRCA1/2* mutational profile in the blood of the CRC patients

Following the exclusion of synonymous mutations, we detected 57 and 140 *BRCA1* and *BRCA2* mutations, respectively, in the blood of CRC patients, suggesting that *BRCA1,* and *BRCA2* mutations in the blood were more prevalent than those found in the tissue of CRC patients. The maximum number of mutations per patient was 25 in the *BRCA1* compared to 59 in the *BRCA2.*

**Fig. 4:**
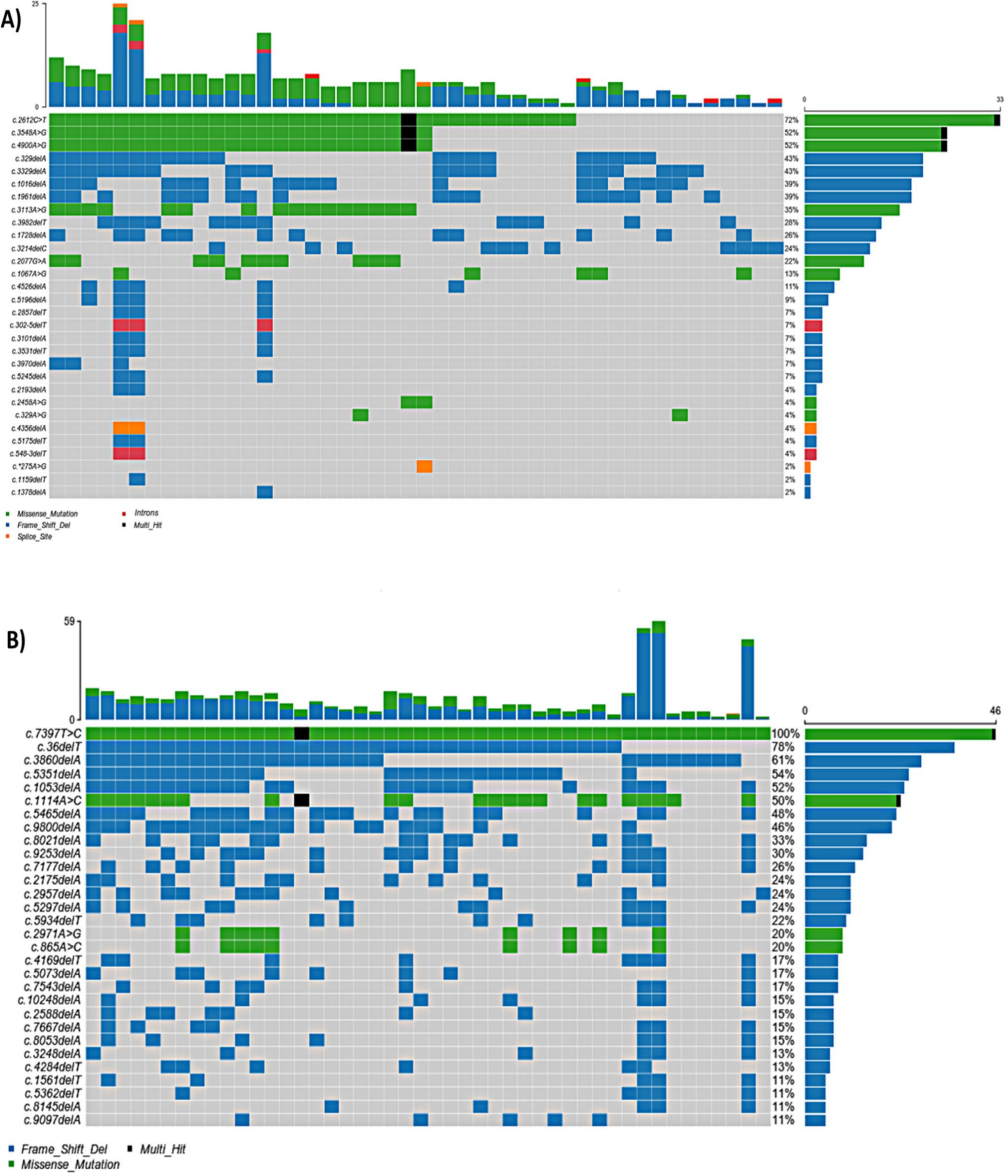
Oncoplots show the overall distribution of highly frequent **A** *BRCA1*, **B** *BRCA2* mutations in the blood of the CRC patients. Each column represents a patient, and each row represents a variant. Different variants colors represent different classifications

The prevailing variant type in both *BRCA1* and *BRCA2* was Del, accounting for 48% and 61%,, followed by SNP at 39% and 38%, respectively. Interestingly, no insertion mutations were identified in either gene within the blood samples, which contrasts with the spectrum of *BRCA1,* and *BRCA2* mutations discovered in the CRC patients' tissue samples (Fig. 4).

Further exploration unveiled that the most recurring SNP type in the *BRCA1* gene was T > C, followed by C > T. In parallel, the primary SNP types in the *BRCA2* gene were T > C trailed by T > G, aligning with the pattern observed in the CRC tissue samples. Figure S3 and Figure S4 depict a comprehensive presentation of the clinical implications and variant classifications for *BRCA1* and *BRCA2* variants, respectively.

**Fig. 5:**
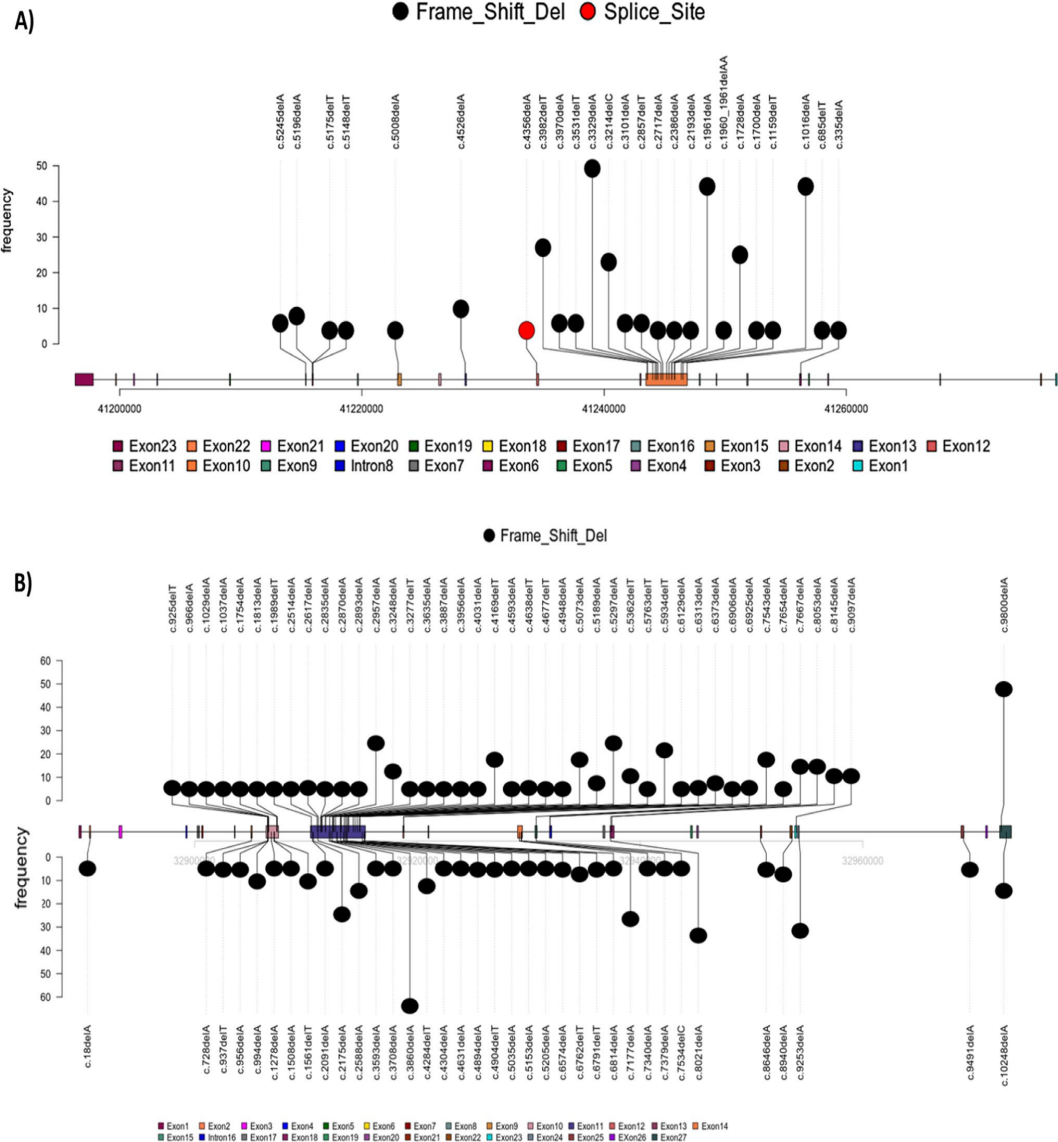
Lollipop representations show the location of the pathogenic mutations in **A** *BRCA1*, **B** *BRCA2* in the blood of the CRC patients. The mutations are colored according to their type

**Fig. 6:**
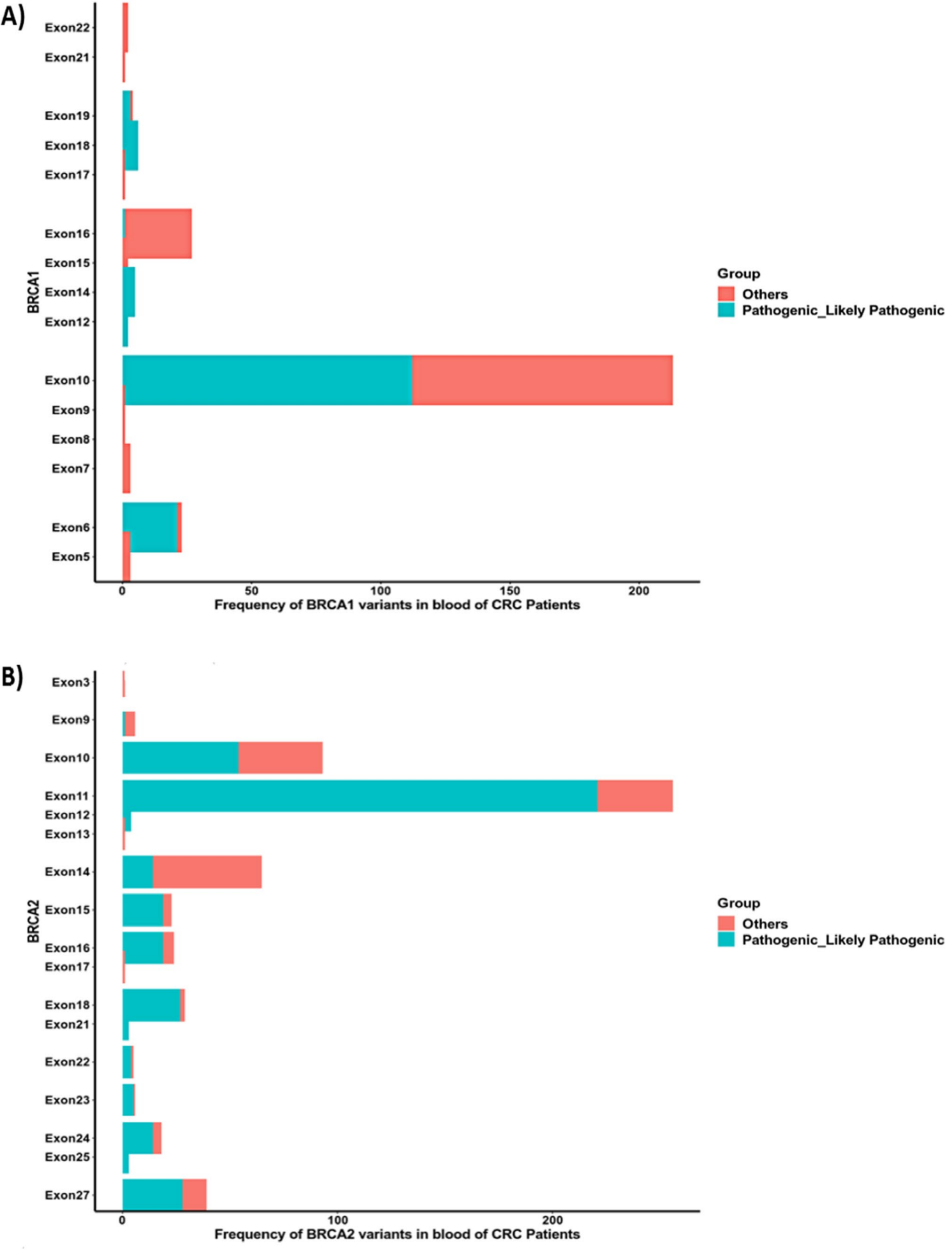
Bar graphs show the most affected exons harbored pathogenic and likely pathogenic mutations in **A** *BRCA1*, **B** *BRCA2* in the blood of the CRC patients. The mutations are colored according to their type

As shown in Figs. 5 and 6, the most affected exons for *BRCA1* PVs were exon 10, followed by exon 6, and the most affected exons for *BRCA2* PVs were exon 11, followed by exon 10, exon 18, and exon 27.

A comparison between the blood samples of CRC patients and control subjects has unveiled specific mutations in *BRCA1* and *BRCA2* genes. Notably, 15 germline CRC-specific mutations were identified in *BRCA1*, while 26 were found in *BRCA2*. Regarding their clinical significance, the germline *BRCA1* mutations were categorized as follows: 12 PVs, 2 VUS, and one LPV.

Notably, two novel PVs (c.3970delA & c.3101delA) were identified in exon 10 of *BRCA1* in 3 patients. The *BRCA2* germline mutations were classified into 9 PVs, 16 VUS, and one LPV. Interestingly, three cases had one novel LPV (c.937delT) in exon 10, two novel PVs (c.2617delA & c.6791delT) in exon 11, and one novel PV (c.9491delA) in exon 25 of *BRCA2* (Table 2).

**Table 2:** Germline *BRCA1* and *BRCA2* mutations detected only in the blood of Egyptian CRC patients

*Gene*	Position	Exon	ID	Type	Clinical Significance	HGVS.c	HGVS.p	CRC Blood (n = 46)	*VMF*
*BRCA1*	Chr17: 41256244	6	rs886040119	DEL	PV	c.335delA	p.Asn112fs	1	0.20
	Chr17 41247925	9	SCV002150654.1	SNP	VUS	c.608A > G	p.Glu203Gly	1	0.17
	Chr17: 41243577	10	SCV002549121.1	DEL	NPV	c.3970delA	p.Met1324fs	3	0.74
	Chr17: 41244016	10	rs80357621	DEL	PV	c.3531delT	p.Phe1177fs	3	0.20
	Chr17: 41244446	10	SCV002549120.1	DEL	NPV	c.3101delA	p.Asn1034fs	3	0.63
	Chr17: 41244830	10	rs876659072	DEL	PV	c.2717delA	p.Lys906fs	1	0.28
	Chr17: 41245586	10	rs80357643	DEL	PV	c.1960_1961delAA	p.Lys654fs	1	0.37
	Chr17: 41245847	10	rs397508899	DEL	PV	c.1700delA	p.Asn567fs	1	0.34
	Chr17: 41246388	10	rs1602208229	DEL	PV	c.1159delT	p.Ser387fs	1	0.05
	Chr17: 41246862	10	rs80357824	DEL	PV	c.685delT	p.Ser229fs	1	0.11
	Chr17: 41234421	12	rs1567782959	DEL	PV	c.4356delA	p.Asp1453fs	2	0.48
	Chr17: 41215909	18	rs730880288	DEL	PV	c.5196delA	p.Lys1732fs	4	0.70
	Chr17: 41215930	18	rs397509228	DEL	PV	c.5175delT	p.Leu1726fs	2	0.58
	Chr17: 41215957	18	rs80357886	DEL	PV	c.5148delT	p.Phe1716fs	1	0.88
	Chr17: 41201176	21	rs1567758993	SNP	VUS	c.5368T > C	-	1	0.25
*BRCA2*	Chr13: 32905126	9	rs587781513	SNP	VUS	c.752C > G	p.Thr251Arg	1	0.51
	Chr13: 32906547	10	SCV002549102.1	DEL	NLPV	c.937delT	p.Ser313fs	3	0.76
	Chr13: 32906565	10	rs80359770	DEL	PV	c.956delA	p.Asn319fs	3	0.23
	Chr13: 32907323	10	rs45564238	SNP	VUS	c.1708A > G	p.Asn570Asp	1	0.12
	Chr13: 32911104	11	SCV002549104.1	DEL	NPV	c.2617delA	p.Ile873fs	3	0.26
	Chr13: 32913381	11	rs1555283980	DEL	PV	c.4894delA	p.Ser1632fs	3	0.52
	Chr13: 32914800	11	rs886040649	DEL	PV	c.6313delA	p.Ile2105fs	3	0.47
	Chr13: 32915279	11	SCV002549114.1	DEL	NPV	c.6791delT	p.Leu2264fs	3	0.62
	Chr13: 32911330	11	rs149753706	SNP	VUS	c.2838T > A	p.Asp946Glu	1	0.12
	Chr13: 32911574	11	rs1567792459	SNP	VUS	c.3082A > G	p.Lys1028Glu	1	0.16
	Chr13: 32912127	11	rs80358609	SNP	VUS	c.3635A > G	p.Asn1212Ser	1	0.16
	Chr13: 32912481	11	rs1057520792	SNP	VUS	c.3989A > G	p.Asn1330Ser	1	0.33
	Chr13: 32912580	11	rs1165204072	SNP	VUS	c.4088A > G	p.Asn1363Ser	1	0.25
	Chr13: 32913272	11	SCV002303336.1	SNP	VUS	c.4780A > G	p.Met1594Val	1	0.27
	Chr13: 32913672	11	rs2072522543	SNP	VUS	c.5180A > G	p.Asn1727Ser	1	0.15
	Chr13: 32915081	11	SCV002171426.1	SNP	VUS	c.6589A > G	p.Thr2197Ala	1	0.50
	Chr13: 32918773	12	rs2072598725	DEL	PV	c.6925delA	p.Ser2309fs	3	0.47
	Chr13 32921012	13	rs80358925	SNP	VUS	c.6986C > T	p.Pro2329Leu	1	0.65
	Chr13: 32937479	18	rs1135401923	DEL	PV	c.8145delA	p.Val2716fs	5	0.45
	Chr13: 32950816	21	rs886040785	DEL	PV	c.8646delA	p.Lys2882fs	3	0.84
	Chr13: 32953515	22	rs755075283	SNP	VUS	c.8816A > G	p.Lys2939Arg	1	0.13
	Chr13 32954049	23	rs80359167	SNP	VUS	c.9116C > T	p.Pro3039Leu	1	0.8
	Chr13: 32954273	24	rs80359190	SNP	VUS	c.9247A > G	p.Lys3083Glu	4	0.12
	Chr13: 32969055	25	SCV002549118.1	DEL	NPV	c.9491delA	p.Asn3164fs	3	0.55
	Chr13: 32972589	27	rs431825381	DEL	VUS	c.9945delA	p.Glu3316fs	1	0.46
	Chr13: 32972782	27	rs1593202359	SNP	VUS	c.10132G > A	p.Asp3378Asn	1	0.38

HGVS.c, Human Genome Variation Society, coding DNA sequence; HGVS.p, Human Genome Variation Society, protein sequence; Chr., Chromosome; CRC, Colorectal Cancer; PV, Pathogenic Variants; LPV, Likely Pathogenic Variant; VUS, Variants of Uncertain Significance; CIP, Conflicting Interpretation of Pathogenicity; NPV, Novel Pathogenic Variant; LNPV, Likely Novel Pathogenic variant; VMF, Variant Major Allele Frequency; SNP, Single Nucleotide Polymorphism; Del, Deletion

It is worth highlighting that among these, two novel PVs (c.3970delA & c.3101delA) were identified in exon 10 of *BRCA1* across 3 patients. As for the *BRCA2* germline mutations, they were classified into 9 PVs, 16 VUS, and one LPV. Interestingly, three cases exhibited unique findings: a novel LPV (c.937delT) in exon 10, two novel PVs (c.2617delA & c.6791delT) in exon 11, and one novel PV (c.9491delA) in exon 25 of *BRCA2* (Table 2).

### Shared *BRCA1/2* PVs in tissue and blood of the CRC patients

Comparing the tissue and blood of CRC patients indicated that 24% of the *BRCA1* and 27% of the *BRCA2* PVs were shared across tissue and blood. *BRCA1* harbored seven PVs, while *BRCA2* harbored 16 PVs, with a higher percentage in the blood than in the tissue.

**Fig. 7:**
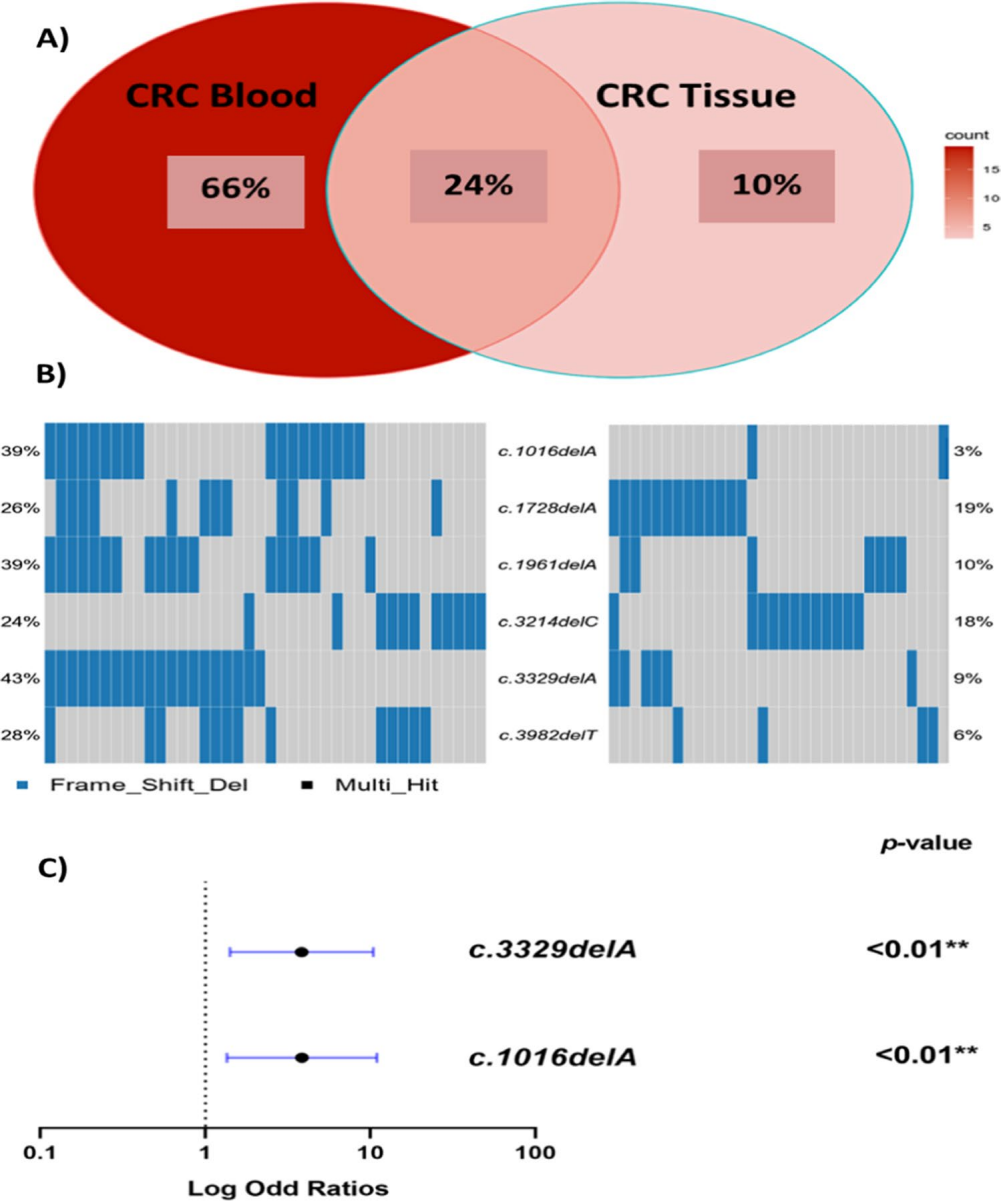
**A** Venn graph displays the percentage of *BRCA1* PVs in both tissue and blood of the CRC patients, **B** Heatmap displays the shared *BRCA1* PVs per patient in the tissue (right side) and the blood (left side) of the CRC patients and **C** Forest plot displays the significant shared *BRCA1* PVs in the CRC blood compared to healthy controls

We demonstrated that six of the seven *BRCA1* PVs shared across the blood and tissue of CRC patients were located in exon10. One of the seven was novel (c.3982delT) and detected in 28% of blood CRC samples but only 6% of tissue CRC samples (*P* = 0.008). When compared to controls, two of seven (c.1016delA & c.3329delA) had significant odds ratios (OR) for CRC risk (OR = 3.5, *P* = 0.0099; OR = 4, *P* = 0.0069, respectively) as illustrated in Table 3, Table S2 and Fig. 7.

**Table 3:** Shared *BRCA1* and *BRCA2* mutations detected in the blood and tissue of Egyptian CRC patients

Gene	Position	Exon	ID	Type	Clinical Significance	HGVS.c	HGVS.p	CRC blood (n = 46)	CRC tissue (n = 82)	*P*-value
*BRCA1*	Chr17:41256250	6	COSM1383528 ;rs80357604	DEL	PV	c.329delA	p.Lys110fs	**20 (43%)**	3 (4%)	< 0.001**
	Chr17: 41244218	10	rs80357575	DEL	PV	c.3329delA	p.Lys1110fs	**20 (43%)**	6 (9%)	< 0.001**
	Chr17: 41245586	10	COSM219054 ;rs80357522	DEL	PV	c.1961delA	p.Lys654fs	**18 (39%)**	7 (10%)	0.001**
	Chr17: 41246531	10	rs80357569	DEL	PV	c.1016delA	p.Lys339fs	**18 (39%)**	2 (3%)	< 0.001**
	Chr17: 41243565	10	SCV001499582.1	DEL	NPV	c.3982delT	p.Ser1328fs	**13 (28%)**	4 (6%)	0.008**
	Chr17: 41245819	10	rs397507192	DEL	PV	c.1728delA	p.Glu577fs	12 (26%)	13 (16%)	0.164
	Chr17: 41244333	10	rs80357923	DEL	PV	c.3214delC	p.Leu1072fs	11 (24%)	12 (18%)	0.193
*BRCA2*	Chr13: 32907171	10	rs886040374	DEL	NPV	c.1561delT	p.Ser521fs	5 (11%)	5 (7%)	0.34
	Chr13: 32906602	10	rs80359777	DEL	PV	c.994delA	p.Ile332fs	5 (11%)	2 (3%)	0.06
	Chr13: 32906888	10	rs80359274	DEL	PV	c.1278delA	p.Asp427fs	2 (4%)	1 (1%)	0.29
	Chr13: 32912345	11	rs80359406	DEL	PV	c.3860delA	p.Asn1287fs	**28 (61%)**	11 (14%)	< 0.001**
	Chr13: 32910661	11	rs276174819	DEL	PV	c.2175delA	p.Val726fs	11 (24%)	3 (4%)	0.002**
	Chr13: 32913783	11	rs1555284157	DEL	PV	c.5297delA	p.Asn1766fs	11 (24%)	5 (7%)	0.006**
	Chr13: 32912655	11	rs80359433	DEL	PV	c.4169delT	p.Leu1390fs	8 (17%)	4 (5%)	0.02*
	Chr13: 32913558	11	COSM1562291; rs80359479	DEL	PV	c.5073delA	p.Lys1691fs	8 (17%)	4 (5%)	0.02*
	Chr13: 32914859	11	rs80359578	DEL	PV	c.6373delA	p.Thr2125fs	4 (9%)	1 (1%)	0.07
	Chr13: 32929161	14	rs397507899	DEL	NPV	c.7177delA	p.Met2393fs	**12 (26%)**	4 (5%)	0.0016*
	Chr13: 32930667	15	rs80359657	DEL	PV	c.7543delA	p.Thr2515fs	8 (17%)	5 (7%)	0.051
	Chr13: 32937354	18	rs397507952	DEL	PV	c.8021delA	p.Lys2674fs	**15 (33%)**	9 (12%)	0.0038*
	Chr13: 32954022	23	COSM1366492; rs397507419	DEL	PV	c.9097delA	p.Thr3033fs	5 (11%)	20 (26%)	0.07
	Chr13: 32954272	24	rs80359752	DEL	PV	c.9253delA	p.Thr3085fs	**14 (30%)**	5 (7%)	0.0007**
	Chr13: 32972445	27	rs1566261027	DEL	NPV	c.9800delA	p.Lys3267fs	**21 (46%)**	5 (7%)	< 0.001**
	Chr13: 32972892	27	COSM309515 ;rs776212316	DEL	NPV	c.10248delA	p.Lys3416fs	7 (11%)	1 (1%)	0.0138*

HGVS.c, Human Genome Variation Society, coding DNA sequence; HGVS.p, Human Genome Variation Society, protein sequence; Chr., Chromosome; CRC, Colorectal Cancer; PV, Pathogenic Variants; LPV, Likely Pathogenic Variant; NPV, Novel Pathogenic Variant; LNPV, Likely Novel Pathogenic variant; VMF, Variant Major Allele Frequency; Del, Deletion

We demonstrated that six of the 16 shared *BRCA2* PVs were located in exon 11. All 16 shared *BRCA2* PVs were more prevalent in the blood than in the tissue of CRC patients, except for c.9097delA, which was more prevalent in the tissue (26% vs. 11%).

Furthermore, we identified four novel shared PVs in exon 10 (c.1561delT), exon 14 (c.7177delA) and exon 27 (c.9800delA & c.10248delA) of *BRCA2* with higher frequency in the blood than the tissue of CRC patients.

**Fig. 8:**
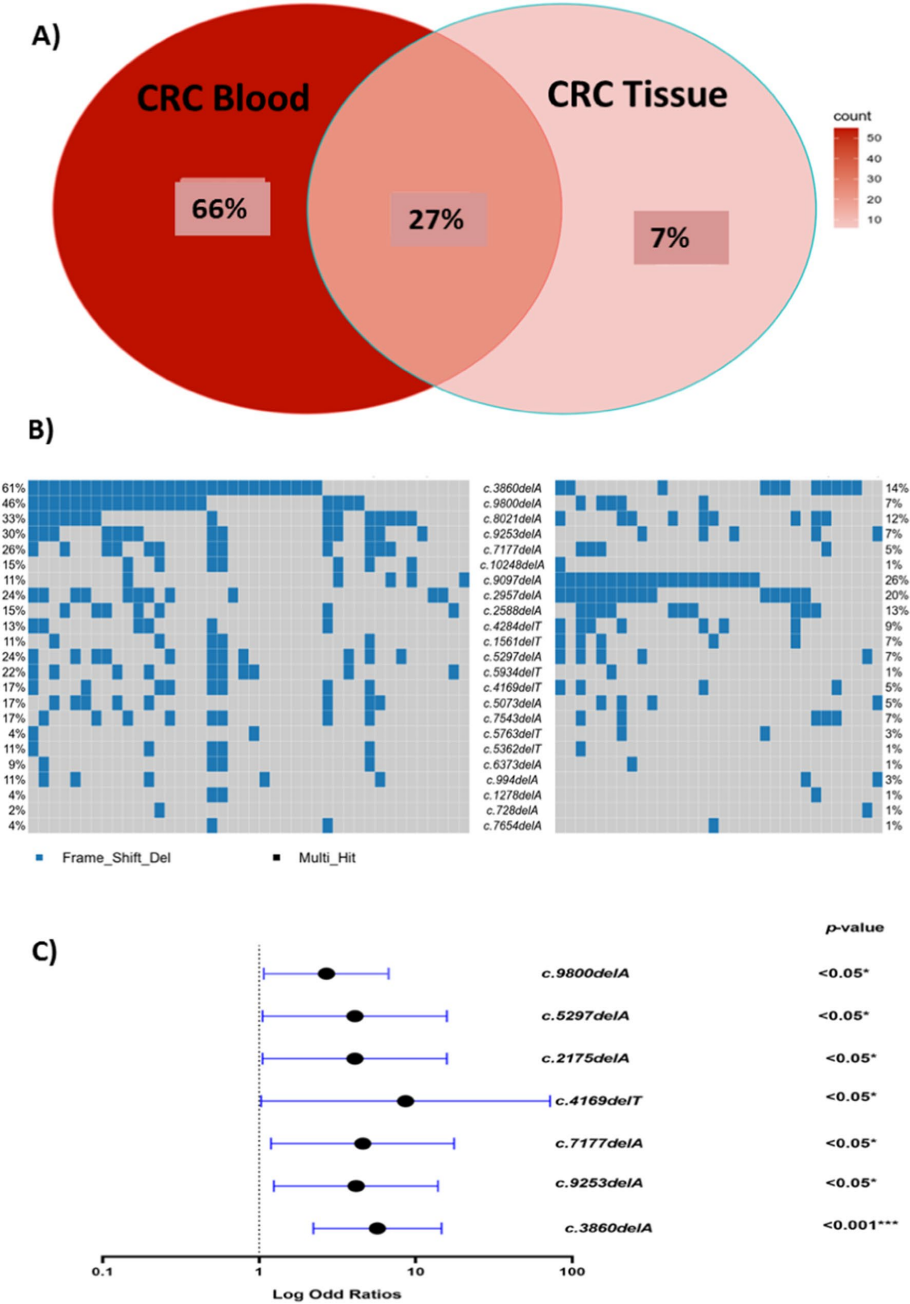
**A** Venn graph displays the percentage of *BRCA2* PVs in both tissue and blood of the CRC patients, **B** Heatmap displays the shared *BRCA2* PVs per patient in the tissue (right side) and the blood (left side) of the CRC patients and **C** Forest plot displays the significant shared *BRCA2* PVs in the CRC blood compared to healthy controls

When we compared shared *BRCA2* PVs to our controls, we discovered that, seven PVs had a significant OR for CRC risk. Four of the seven PVs (c.2175delA, c.3860delA, c.4169delT, and c.5297delA) were found in exon11 (OR = 4.2, *P* = 0.038; OR = 5.8, *P* = 0.0002; OR = 8.8, *P* = 0.04; OR = 4.2, *P* = 0.038, respectively); one (c.7177delA) was found in exon14 (OR = 4.7, *P* = 0.02), one (c.9253delA) was found in exon 24 (OR = 4.3, *P* = 0.01) and one (c.9800delA) was found in exon 27(OR = 2.7, *P* = 0.02) as illustrated in Table 3, Table S2 and Fig. 8.

### Co-occurring and mutually exclusive events between *BRCA1* and *BRCA2* PVs

There are 24 co-occurring events of *BRCA1/2* pathogenic variant pairs in CRC patients' tissue. Among these, 16 events were found between distinct *BRCA2* variants, two events between various *BRCA1* variants, and 6 events involving combinations of *BRCA1* and *BRCA2* variants.

Among the co-occurring events associated with *BRCA2* variants, two notable pairs were c.9097delA & c.9800delA (*P* = 0.01, event ratio = 5/15) and c.9097dupA & c.5566 5567delCAinsTG (*P* = 0.04, 1/1). Co-occurring events between *BRCA1* variants include c.3329delA & c.1728delA (*P* = 0.04, 9/5), as well as c.3329dupA &c.1961dupA (*P* = 0.04, 1/1).

**Fig. 9:**
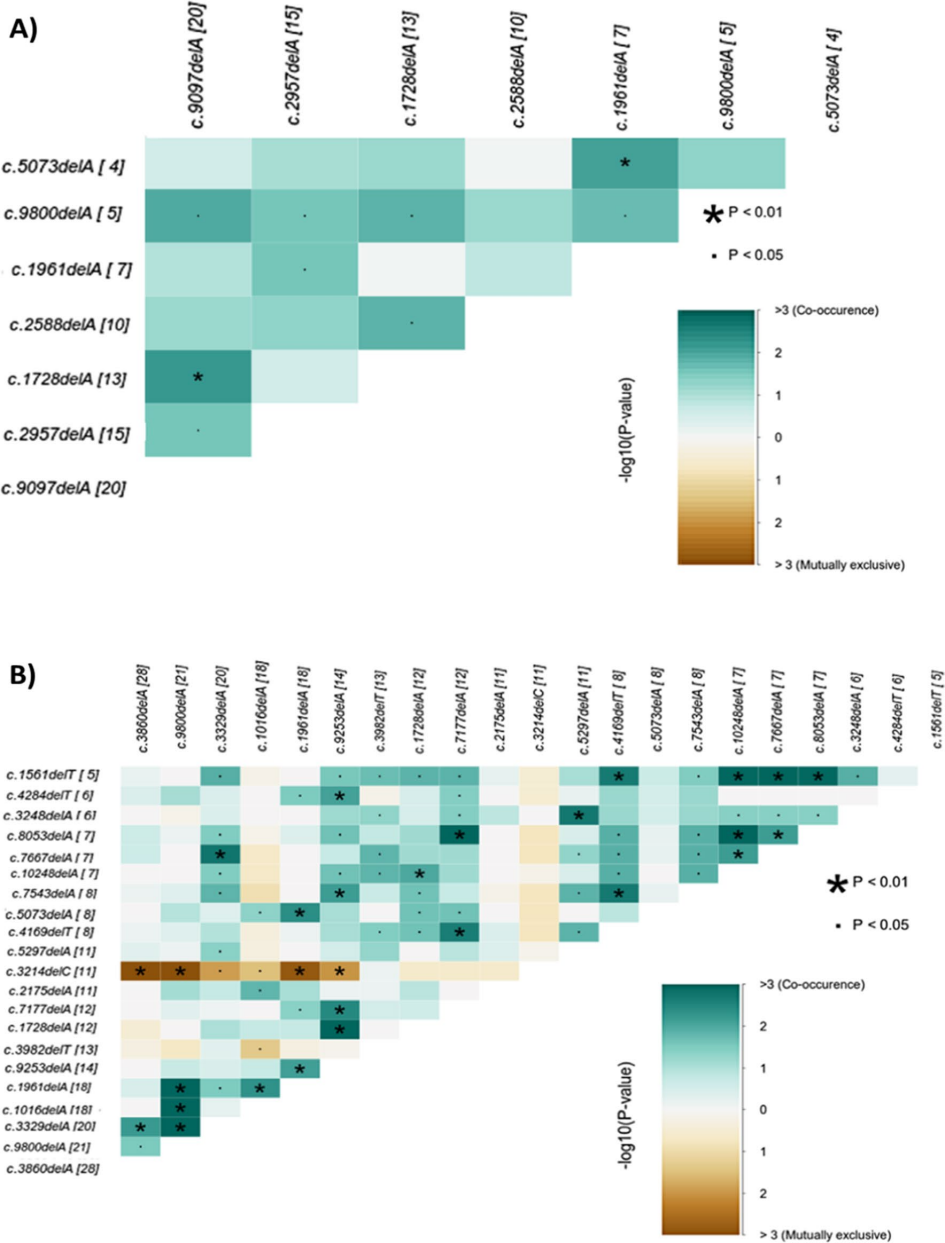
Heatmap plot shows the variant pairs with co-occurring and mutually exclusive events of *BRCA1* and *BRCA2* PVs in **A** the CRC tissues and **B** the CRC blood. The significance level is indicated in the legend

Furthermore, instances of *BRCA1* and *BRCA2* co-occurring events encompassed c.1728delA with c.9097delA and c.9800delA (*P* = 0.006, 13/10, and *P* = 0.01, 10/4, respectively) and c.1961delA with c.5073delA and c.9800delA (*P* = 0.008, 5/3 and *P* = 0.01, 6/3, respectively) as shown in Supplementary Table 2 and Fig. 9a.

Turning to the blood samples from CRC patients, a total of 71 co-occurring events and 7 mutually exclusive events involving pairs of *BRCA1/2* pathogenic variants were observed. Among these, 38 events were identified within *BRCA2* variants, two events within various *BRCA1* variants, and 31 events between *BRCA1* and *BRCA2* variants. Prominent examples of co-occurring events associated with *BRCA2* variants included c.7177delA with c.5073delA, c.4169delT, and c.9253delA (*P* = 0.02, 5/10; *P* = 0.002, 8/6; and *P* = 0.003, event ratio = 10/8), c.8021delA & c.9253delA (*P* = 0.03, 8/13), and c.3860delA & c.9800delA (*P* = 0.02, 7/15). Furthermore, instances of co-occurring events between *BRCA1* variants were c.1961delA with c.1016delA and c.3329delA (*P* = 0.004, 12/12 and *P* = 0.03, 12/14).

In addition, the co-occurring events between *BRCA1* and *BRCA2* variants were c.1961delA with c.7177delA, c.9253delA, c.5073delA and c.9800delA (*P* = 0.04, 14/8; *P* = 0.0007, 12/10; *P* = 0.004, 12/7 and *P* = 3.43606E-06, 7/16, respectively). Also, the co-occurrence between c.3329delAwith c.3860delA and c.9800delA (*P* = 0.006, 14/17 and *P* = 0.0009, 11/15, respectively). Additionally, co-occurrence between c.1016delA, and c.9800delA (*P* = 0.0008, 11/14).

There were four mutually exclusive events within *BRCA1* variants and three events between *BRCA1* and *BRCA2* variants. The most important significant mutually exclusive events between *BRCA1* variants were c.1016delA with c.3982delT (*P* = 0.04, 2/27) and c.3214delC with c.1016delA, c.3329delA and c.1961delA (*P* = 0.03, 1/27;* P* = 0.01, 1/29 and *P* = 0.001, 0/29, respectively).The mutually exclusive events between *BRCA1* and *BRCA2* variants included c.3214delC with c.9253delA, c.9800delA, and c.3860delA (*P* = 0.009, 0/25;* P* = 0.0002, 0/32 and *P* = 5.48657E-05, 1/37, respectively) as shown in Table S4 and Fig. 9b.

### Ethnic-related variants of *BRCA1/2* in our cohort

We have identified six highly prevalent SNPs in both the CRC and control groups when compared to other populations; four in the *BRCA1* gene and two in the *BRCA2* gene.

Regarding *BRCA1* SNPs, we found that the major allele frequency (VMF) of c.4900A > G, c.3548A > G, and c.3113A > G exhibited stronger resemblances to the South-Asian population’s VMF, whereas VMF of c.2612C > T was more related to that of the African population. Correlation analysis between ethnic-related *BRCA1* SNPs and *BRCA1* PVs in the CRC patients’ blood revealed that there was a positive correlation between c.3548A > G SNP and c.5196delA germline PV (r = 0.3, *P* < 0.05).

Turning to the *BRCA2* SNPs, we revealed that the VMF of c.7397 T > C and c.1114A > C in our cohort resembled the VMF observed in the South-Asian population. Moreover, the correlation analysis between ethnic-related *BRCA2* SNPs and *BRCA2* PVs in the CRC patients’ blood revealed that there were positive correlations between c.7397 T > C SNP and c.3860delA PV (r = 0.3, *P* < 0.05), as well as c.1114A > C SNP with c.4169delT and c.7177delA PVs (r = 0.5, *P* < 0.05; r = 0.3, *P* < 0.05, respectively).

We noticed four frameshift *BRCA2* variants in our controls with an occurrence rate > 30%. The c.36delT and c.5351delA were detected in 65% and 33% of the controls, respectively, and were identified as PVs based on the ClinVar database and CADD score, which were the other two variants (c.5465delA and c.1053delA) detected in 47% and 33% of the controls, respectively, were identified as novel variants. The CADD score suggested their potential pathogenicity, as outlined in Table 4.

**Table 4:** Ethnic–related *BRCA1/2* variants detected in the blood of the Egyptian controls in comparison with other populations

Gene	Position	Exon	ID	HGVS.c	HGVS.p	Type	Clinical Significance	SIFT	CADD	Control (n=43)	Our VMF	East Asia VMF	South Asia VMF	African VMF	Europe VMF	LatinoVMF	Jewish VMF
*BRCA1*	Chr17: 41223094	16	rs1799966	c.4900A > G	p.Ser1634Gly	SNP	VUS	Tolerated	< 10	29 (74%)	0.64	0.37	0.50*	0.23	0.34	0.32	0.37
	Chr17:41244936	10	rs799917; COSM148278	c.2612C > T	p.Pro871Leu	SNP	Benign	Activating	18.04	26 (67%)	0.70	0.37	0.53	0.82*	0.34	0.35	0.37
	Chr17: 41244000	10	rs16942; COSM148277	c.3548A > G	p.Lys1183Arg	SNP	Benign	Tolerated	< 10	26 (67%)	0.70	0.37	0.50*	0.23	0.34	0.32	0.37
	Chr17: 1244435	10	rs16941	c.3113A > G	p.Glu1038Gly	SNP	Benign	Damaging	15.07	13 (33%)	0.48	0.27	0.35*	0.12	0.27	0.30	0.27
*BRCA2*	Chr13: 32929387	14	rs169547	c.7397T > C	p.Val2466Ala	SNP	Benign	Tolerated	11.56	40 (93%)	1.00	0.998	0.999*	0.929	0.997	0.988	0.998
	Chr13: 32906729	10	rs144848; COSM147663	c.1114A > C	p.Asn372His	SNP	Benign	Tolerated	12.8	22 (51%)	0.58	0.269	0.353*	0.121	0.270	0.299	0.269
	Chr13: 32890627	2	rs80359399	c.36delT	p.Phe12fs	DEL	PV	–	29.5	28 (65%)	0.61	–	–	–	–	–	–
	Chr13: 32906663	10	rs886040342	c.1053delA	p.Lys351fs	DEL	NPV	–	21.5	14 (33%)	0.59	–	–	–	–	–	–
	Chr13: 32913952	11	rs1555284237	c.5465delA	p.Asn1822fs	DEL	NPV	–	22.5	20 (47%)	0.38	–	–	–	–	–	–
	Chr13: 32913836	11	COSM18607 ;rs80359509	c.5351delA	p.Asn1784fs	DEL	PV	–	20.5	14 (33%)	0.46	–	–	–	–	–	–

HGVS.c, Human Genome Variation Society, coding DNA sequence; HGVS.p, Human Genome Variation Society, protein sequence; Chr., Chromosome; CRC, Colorectal Cancer; PV, Pathogenic Variants; NPV, Novel Pathogenic Variant; VMF, Variant Major Allele Frequency; Del, Deletion

### Prevalence of HPV and its correlation with *BRCA1/2* PVs and VUS in the tissue of CRC patients

**Fig. 10:**
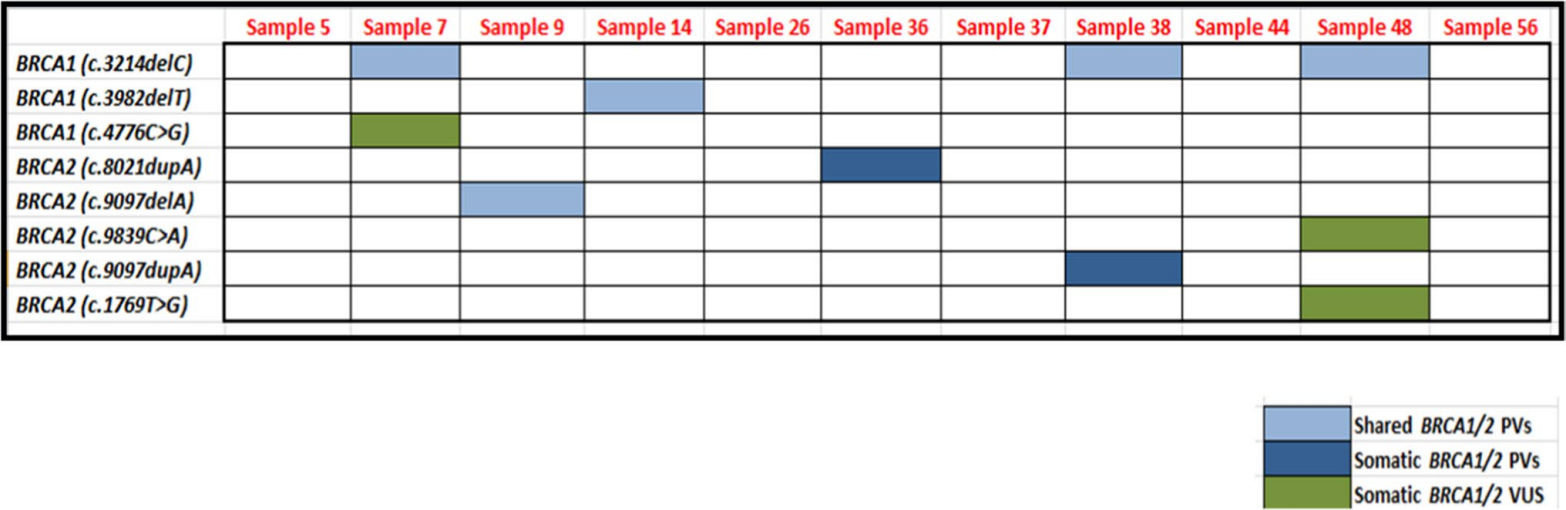
Schematic diagram shows the distribution of *BRCA1*/*2* PVs and VUS in the HPV-positive CRC tissues. The color code is indicated in the legend

Our results revealed that 15 of the 64 CRC tissues (23.8%) tested positive for Human Papillomavirus (HPV) infection. Moreover, eleven of the 15 HPV-positive cases had *BRCA1/2* mutations. Regarding *BRCA1/2* PVs and VUS in HPV-positive cases, there were six cases of the eleven (54%) harbored three and five PVs in *BRCA1* and *BRCA2* genes, respectively. The distribution of *BRCA1/2*PVs and VUS in the CRC patients is illustrated in Fig. 10.

## Discussion

One of the major worldwide causes of morbidity and mortality is colorectal cancer (CRC)[1]. To our knowledge, this is the initial investigation utilizing high-throughput genomic sequencing to analyze the mutational patterns of *BRCA1* and *BRCA2* in both tissue and blood samples of Egyptian CRC patients. This novel approach highlights the significance of these genetic factors in CRC development and progression. Moreover, the exploration of the potential correlation between HPV infection and *BRCA1/2* mutations, along with the assessment of ethnic-specific polymorphisms of *BRCA1* and *BRCA2* in healthy Egyptian controls to those of other populations, adds a layer of depth to this investigation.

Our principal findings revealed that *BRCA1/2* mutant carriers had an elevated risk of developing CRC, suggesting a potential link between these mutations and CRC. Furthermore, the identification of germline variations highlights the possibility of hereditary origins for these mutations, supporting the notion of hereditary susceptibility to CRC. Furthermore, we detected HPV infection in 23.8% of CRC patient tissues, and more than half of the HPV-positive cases co-occurred with *BRCA1/2* PV, suggesting its potential role in CRC in Egypt, along with *BRCA1/2* PVs.

Our findings showed that the CRC group had a higher prevalence of *BRCA1/2* mutations compared to the control group. The application of pathway analysis utilizing IVA has revealed pervasive loss-of-function activity associated with *BRCA1/2* mutations in the CRC group; indicating the potential role of theS *BRCA* damage pathway in colorectal carcinogenesis.

Existing literature presents a spectrum of evidence concerning the correlation between *BRCA1/2* mutations and CRC susceptibility. In contrast to our study, which has yielded substantial insights into the potential implications of *BRCA1/2* mutations in the context of CRC, other investigations proposed a modestly increased CRC risk in *BRCA1* carriers. Conversely, evidence of CRC risk among *BRCA2* carriers remains lacking [26–28].

Discrepancies among these studies can be attributed to variations in sample sizes, demographic traits, study designs, variant classifications, and potential confounding variables such as age, lifestyle choices, and concurrent genetic mutations. The mismanagement of these factors has contributed to the observed variability. However, Sopik et al. [29] employed a comprehensive research approach encompassing various study types, suggesting an increased CRC risk within individuals carrying *BRCA1/2* mutations in high-risk familial contexts.

Interestingly, a notable contrast in prevalence has emerged between *BRCA1* and *BRCA2* mutations in the current study. Specifically, a higher prevalence of *BRCA2* mutations has been detected indicating the distinctive roles of *BRCA2* in the context of CRC. Our observation contrasts with previous research findings. Notably, a systematic meta-analysis study [26] highlighted a moderate rise in CRC risk, specifically among *BRCA1* mutation carriers. Pivoting the nuances of methodology, our study diverges from prior research conducted by Phelan et al. [30] which explored a significant cohort of women with *BRCA1* and *BRCA2* mutations. The authors revealed an intriguing 4.76-fold surge in risk among women aged 30–49 years with the *BRCA1* mutations.

On the other hand, a previous research demonstrated a higher prevalence of *BRCA2* mutations compared to *BRCA1* in other cancer types, including populations at increased risk for hereditary breast and ovarian cancer [31–33].

Notably, the absence of studies on the Egyptian population and the exclusive focus on high-risk families in such studies underscore the unique contribution of our research. Conducted within the Egyptian population, our study adds an ethnic dimension, augmenting the importance of our results. In essence, our research bridges gaps by exploring genetic factors, population attributes, and familial predisposition in CRC risk.

Intriguingly, our investigation revealed a higher prevalence of *BRCA1/2* mutations in blood samples compared to tissue samples. This indicates that blood might be a valuable source of genetic information, offering a less invasive way to study cancer-related mutations. Consistent with findings by Szczerba et al. [34], who reported a low incidence of somatic PVs in *BRCA1/2* mutations in Polish patients with breast cancer, our research also revealed a relatively low incidence of somatic PV within the *BRCA1/2* genes, ranging from 1% to 2.5%, in CRC patients. This congruence in observations across cancer types and populations highlights the rarity of these mutations in certain cohorts.

This study brings forth intriguing findings regarding the distribution of *BRCA1/2* germline PVs. Notably, these variants were prominently located in exon 10 for *BRCA1* and exon 11 for *BRCA2,* mirroring the distribution pattern observed for *BRCA1/2* somatic PVs. This underscores the susceptibility of the relatively larger exon 10 and exon 11 in both genes to heightened mutational activity, as previously suggested by Darabi et al. [35].

As previously stated, our data revealed a significant disparity between the incidence of shared *BRCA1/2* PVs in the blood and tissue of our CRC patients. Notable among these variants are the *BRCA1* PVs, encompassing c.329delA, c.3329delA, c.1016delA, and c.1961delA variants. Of note, we demonstrated that c.329delA increases the risk of CRC by 2.5-fold compared to healthy controls. Intriguingly, the pathogenicity of this variant has been highlighted as related to pancreatic cancer [36]**.** Another shared *BRCA1* PV (c.3329delA) displays a fourfold elevation in CRC risk. The prevalence of this variant in breast and ovarian cancers among African Americans [37], Brazilians [38], and the Chinese population [39] has been well-documented, suggesting its broader implications.

Furthermore, we demonstrated that individuals harboring the c.1016delA mutation exhibit a 3.5-fold elevated risk of developing CRC compared to the controls. This observation is consistent with prior research attributing significance to this mutation in hereditary cancer syndromes. Its detection across diverse populations, including Belgium [40, 41], India [42], Vietnam [43] and Japan populations [44], through various techniques like high-resolution melt curve (HRM) real-time PCR and next-generation sequencing (NGS), underscores its global relevance.

Similarly, the c.1961delA mutation is associated with a 2.8-fold rise in CRC risk when compared to controls. Notably, this mutation's implications extend to breast, ovarian, and hereditary cancer syndromes, as observed in South Korea [45], Baltic [46] and Brazil populations [47].

Remarkably, the c.1961delA variant demonstrates a noteworthy co-occurrence with the c.1016delA and c.3329delA variants, suggesting potential interactions or relationships among *BRCA1* PVs that could influence disease development or other genetic outcomes. The observed significant association between specific somatic *BRCA1* PVs namely, c.1961dupA and c.3329dupA in CRC patients in our results align with previous studies that have documented significant associations among these *BRCA1* PVs not only in breast cancer cases [46, 48] but also in ovarian cancer contexts [49, 50]**.** This suggests a potential implication of these co-occurring PVs across different malignancies, pointing towards potentially underlying mechanisms or vulnerabilities linked to these genetic alterations.

On the other hand, we identified two additional *BRCA1* PVs (c.1728delA and c.3214delC) that are equally prevalent in both CRC patient tissue and blood. Interestingly, c.3214delC has been associated with multiple primary cancers [51], as well as breast, ovarian [52], and pancreatic cancers [36] in the Asian population. In contrast, c.1728delA has been relatively sparingly reported.

In the realm of *BRCA2* variants, an additional cluster of five PVs (c.3860delA, c.2175delA, c.5297delA, c.4169delT, and c.5073delA) emerged from our investigation. Positioned on exon 11, these variants displayed a distinctive prevalence pattern, with markedly elevated frequencies in blood samples in contrast to their tissue counterparts within the CRC patient cohort. Crucially, these mutations exhibited a robust correlation with an increased likelihood of CRC development. Prior research has similarly highlighted the significance of these variants in the pathogenicity and development of a spectrum of cancers, spanning breast, ovarian, and hepatocellular carcinoma, among diverse populations [53–61].

Furthermore, we identified two specific *BRCA2* PVs (c.8021delA and c.9253delA) with also an enhanced presence in the blood samples of CRC-diagnosed individuals compared to corresponding tissue samples. The shared c.8021delA variant displayed a 2.9-fold increased risk of CRC. Extensive literature has probed into the pathological implications of c.8021delA, especially within breast and ovarian cancer contexts, across distinct populations such as Chinese [62] and Argentinians [63].

Additionally, we showcased that CRC patients harboring c.9253delA exhibited a 4.3-fold heightened risk of CRC development. Previous studies have delved into the detrimental consequences of this variant within breast and ovarian cancer, focusing on populations such as Romanian [64] and Chinese [65]. Moreover, a noteworthy observation emerged from our investigation; revealing the consequential co-occurrence of c.8021delA and c.9253delA among our CRC patient cohort. This intriguing correlation highlights the potential synergistic implications of these variants in the pathogenesis of CRC.

In the present study, three novel *BRCA2* PVs (c.7177delA, c.9800delA, and c.10248delA) were ascertained shared among both blood and tissue samples from CRC patients. Of significance, a distinct prevalence pattern surfaced, with these variants exhibiting markedly higher frequencies in CRC patient blood samples than in tissue samples. Importantly, c.7177delA and c.9800delA mutations were correlated with an increased propensity to develop CRC. This observation strengthens the notion that these specific genetic alterations may contribute to the susceptibility of individuals to develop CRC, potentially functioning as risk indicators.

Furthermore, our investigation highlighted significant co-occurrences between the novel PV c.7177delA and other shared *BRCA2* PVs (c.4169delT, c.5073delA, and c.9253delA). Notably, the newly identified variant c.9800delA exhibited significant co-occurrence with the highly prevalent shared *BRCA2* PV c.3860delA. These findings collectively point towards a synergistic role between these novel variants and other pathogenic variants in the context of CRC development. These co-occurrence patterns suggest potential synergistic interactions or cumulative effects between these novel variants and other PVs, implying a more complex interplay in the genetic predisposition to CRC development.

While the simultaneous presence of PVs in two distinct cancer-associated genes is uncommon [66], our study's unexpected discovery of 31 co-occurring *BRCA1/2* variants challenges this notion. These findings imply an intricate interplay between *BRCA1* and *BRCA2* PVs in our CRC patient cohort, without significantly amplifying cancer risk or introducing distinct phenotypic characteristics beyond what would be expected from an individual *BRCA* variant. This observation raises the possibility that individuals with concurrent variations might benefit from tailored surveillance programs, intensified screening measures, or specialized risk reduction strategies that deviate from standard protocols based on single PVs. Intriguingly, we also identified mutually exclusive events involving *BRCA1* PV c.3214delC and the widely shared *BRCA2* PVs (c.3860delA, c.9253delA, and c.9800delA). This phenomenon suggests an independent occurrence of *BRCA1* PV c.3214delC, apart from the influences of *BRCA1* or *BRCA2* PVs.

Exploring prevalent mutations within our control group, which have been hypothesized as founder mutations within the Egyptian population, we conducted a comparative analysis with other populations. We detected distinct clusters of SNPs in both the *BRCA1* and *BRCA2* genes. Remarkably, these SNP clusters demonstrated associations with South Asian ethnicity, except for c.2612C > T, which exhibited stronger links to African ethnicity according to the ExAC (genome AD) database [21]**.** This intriguing ethnic diversity indicates Egypt's unique genetic composition, situated as a transcontinental nation between the northeastern corner of Africa and the southwestern corner of Asia. Notably, previous research corroborates these findings, as the identified *BRCA1* (c.4900A > G, c.2612C > T, c.3548A > G, 0.3113A > G) and *BRCA2* (c.7397 T > C, c.1114A > C) clusters were observed in an earlier Egyptian study [67] as well as within Arab African populations like Algerian [68], Bahrainian [69], Moroccan [70] and Tunisian [71] groups, alongside Asian populations encompassing Chinese [72], Korean [73], Iranian [74] and Indian [75] cohorts, and even in American and European populations such as Brazilian [76], and Italian [77] ones, respectively. This reinforces the intricate interplay of various ethnic backgrounds shaping the genetic landscape of Egypt. The correlation analysis in our study indicated significant associations between ethnic-related variants and pathogenic *BRCA1/2* variants, further highlighting a potential relationship between the identified pathogenic *BRCA1/2* variants and specific Egyptian ethnic groups.

Intriguingly, our analysis revealed the presence of four frameshift mutations in *BRCA2* among our control samples, exhibiting frequencies ranging from 33 to 65%. While 2 of these mutations (c.36delT, c.5351delA) were well-established as pathogenic according to the ClinVar database, the remaining two (c.1053delA, c.5465delA) were novel and demonstrated pathogenic potential based on CADD score [24] predictions. Its high frequency in our population might be explained by the fact that such mutations had little or no influence on our population. Also, the Egyptian population's genetic makeup differs from other populations and has various ethnicities. Thus, our study highlights the significance of utilizing our control group as a reference for detecting mutations, given the nuanced genetic landscape that characterizes the Egyptian population.

Moreover, we observed HPV infection in 23.8% of CRC patient tissue samples in accordance with previous Egyptian publications [7, 8]. Hafez et al. detected HPV infection in 22% of CRC patient tissue samples using immunohistochemistry [7], and Sheikh et al. detected the same percentage of HPV infection in breast cancer (BC) patient’s tissue using Real-time PCR [8]. An earlier review also highlighted the carcinogenic significance of HPV in the development of CRC. This review addressed that patients with HPV infection had three times more likely susceptibility to develop CRC [78]. This consistency highlights the involvement of HPV infection in the development and progression of CRC among the Egyptian population.

Genomic instability in widely produced tumors may result from harm to DNA checkpoint suppression, viral replication stress-driven DNA damage, amplification and structural organization of integrated viral DNA, or any combination of these processes. Additionally, research on HPV-related malignancies has revealed that random integration into DNA repair genes was discovered, adding another element causing genomic instability [79, 80]. Through rearrangements between integrated copies, replication of integrated HPV genomes may potentially promote focal genomic instability [5].

Furthermore, our study revealed that more than half of the HPV-positive CRC cases were found in association with pathogenic somatic mutations; *BRCA2* c.8021dupA and c.9097dupA, as well as pathogenic shared mutations; *BRCA1* c.3214delC and c.3982delT. Additionally, HPV was found in combination with two somatic *BRCA2* variants with uncertain significance; c.1769 T > G and c.9839C > A. Thus, our data suggested the potential role of HPV in Egyptian patients with CRC, as well as its association with *BRCA1/2* PVs in the tissue of the CRC patients.

## Conclusion

We conclude that *BRCA1/2* genes are highly mutated in Egyptian CRC patients, especially those with HPV infection. These findings suggest that HPV infection may play a role in the development of CRC in Egypt, particularly in co-occurrence with *BRCA1/2* PVs, which might benefit CRC patients with personalized treatment. Liquid biopsies are more representative than tissue biopsies for *BRCA1/2* mutations, with *BRCA2* mutations occurring at double the incidence of *BRCA1* mutations, indicating that *BRCA1/2* mutations may be readily detected in CRC patients' blood samples. Furthermore, the identified mutation hotspots in exons 6 and 10 of *BRCA1*, and exons 11, 14, 18, 24, and 27 of *BRCA2* are the most impacted, respectively. Since the Egyptian population's genetic composition differs from other populations, we should use our healthy controls as a reference for genetic mutations to differentiate between pathogenic variants causing disease and ethnic -related variants. Our ethnic-related variants are closer to South Asian than African. These discoveries establish the framework for future cancer care innovations and advancements, fostering a holistic understanding of the genetic and viral factors influencing CRC development and progression.

## Limitations

Expanding the tissue sample size among CRC patients is necessary to confirm the potential correlation between HPV infection and *BRCA1/2* mutations. Furthermore, investigating other DNA damage repair genes (DDR) could uncover possible correlations with HPV infection in CRC patients. Additionally, augmenting the blood sample size of CRC patients is recommended to validate our findings related to the novel PVs identified within *BRCA1/2* genes.

## Recommendation and future prospective

We suggest employing High-Resolution Melting Curve (HRM) real-time PCR as an affordable method to validate the extremely frequent pathogenic *BRCA1/2* mutations in the most impacted exons in the blood of Egyptian CRC patients and their families as a non-invasive sample for cancer screening. Moreover, sequencing of HPV-positive cases is highly recommended to identify the genotypes associated with CRC patients in Egypt. Additionally, survival analysis and its correlation with *BRCA1/2* PVs and HPV infection are essential to elucidate its role as a prognostic factor and develop a personalized treatment (Platinum-based drugs) that efficiently targets *BRCA1/2* mutations. Further research is needed to fully understand these findings' clinical implications and determine the optimal course of treatment for individuals with *BRCA1/2* mutations.

### Supplementary Information

Below is the link to the electronic supplementary material.

Supplementary file1 (DOCX 5676 KB)

## Data Availability

All data generated or analyzed during this study and its supplementary information files are included in this article. Moreover, the novel variants predicted to be deleterious were submitted to the ClinVar submission portal (public repository), **(**Organization ID: 507536; Genomic Center, National Cancer Institute, Egypt); Clinvar Link: https://www.ncbi.nlm.nih.gov/clinvar/submitters/507536/.

## References

[1] WHO - World Health Organization (2020). GLOBOCAN - colorectal cancer incidence in the world. Glob. Cancer Obs..

[2] Ibrahim AS, Khaled HM, Mikhail NN, Baraka H, Kamel H (2014). Cancer incidence in Egypt: results of the national population-based cancer registry program. J Cancer Epidemiol.

[3] Meyerson W, Meyerson W, Leisman J, Navarro FCP, Navarro FCP, Gerstein M, Gerstein M, Gerstein M, Gerstein M (2020). Origins and characterization of variants shared between databases of somatic and germline human mutations. BMC Bioinf.

[4] de Martel C, Georges D, Bray F, Ferlay J, Clifford GM (2020). Global burden of cancer attributable to infections in 2018: a worldwide incidence analysis. Lancet Glob Heal.

[5] Weitzman MD, Fradet-Turcotte A (2018). Virus DNA replication and the host DNA damage response. Annu Rev Virol.

[6] Santos N, Tocantins PDB, Leão-Cordeiro JAB, Ataides FS, De Oliveira Ros Maarques L, Silva AMTC (2022). The human papillomavirus in colorectal cancer. J Med Sci.

[7] Hafez FS, Meckawy GR, Alorabi M, Shakweer MM (2022). Interpretation of P16 expression as a marker of HPV in colorectal cancer. Histol Histopathol.

[8] El-Sheikh N, Mousa NO, Tawfeik AM, Saleh AM, Elshikh I, Deyab M, Ragheb F, Moneer MM, Kawashti A, Osman A (2021). Assessment of human papillomavirus infection and risk factors in Egyptian women with breast cancer. Breast Cancer Basic Clin Res.

[9] AlDubayan SH, Giannakis M, Moore ND, Han GC, Reardon B, Hamada T, Mu XJ, Nishihara R, Qian Z, Liu L (2018). Inherited DNA-repair defects in colorectal cancer. Am J Hum Genet.

[10] Moretto R, Elliott A, Zhang J, Arai H, Germani MM, Conca V, Xiu J, Stafford P, Oberley M, Abraham J (2022). Homologous recombination deficiency alterations in colorectal cancer: clinical, molecular, and prognostic implications. J Natl Cancer Inst.

[11] Huyghe JR, Bien SA, Harrison TA, Kang HM, Chen S, Schmit SL, Conti DV, Qu C, Jeon J, Edlund CK (2019). Discovery of common and rare genetic risk variants for colorectal cancer. Nat Genet.

[12] Li LY, Guan YD, Chen XS, Yang JM, Cheng Y (2021). DNA repair pathways in cancer therapy and resistance. Front Pharmacol.

[13] Díez-Villanueva A, Sanz-Pamplona R, Solé X, Cordero D, Crous-Bou M, Guinó E, Lopez-Doriga A, Berenguer A, Aussó S, Paré-Brunet L (2022). COLONOMICS - integrative omics data of one hundred paired normal-tumoral samples from colon cancer patients. Sci Data.

[14] Molinaro E, Andrikou K, Casadei-Gardini A, Rovesti G (2020). BRCA in gastrointestinal cancers: current treatments and future perspectives. Cancers (Basel).

[15] Daly MB, Pal T, Berry MP, Buys SS, Dickson P, Domchek SM, Elkhanany A, Friedman S, Goggins M, Hutton ML (2021). Genetic/familial high-risk assessment: breast, ovarian, and pancreatic, version 2.2021. JNCCN J Natl Compr Cancer Netw.

[16] Zimmer K, Kocher F, Puccini A, Seeber A (2021). Targeting BRCA and DNA damage repair genes in GI cancers: pathophysiology and clinical perspectives. Front Oncol.

[17] Alhusaini A, Cannon A, Maher SG, Reynolds JV, Lynam-Lennon N (2021). Therapeutic potential of parp inhibitors in the treatment of gastrointestinal cancers. Biomedicines.

[18] Golan T, Hammel P, Reni M, Van Cutsem E, Macarulla T, Hall MJ, Park J-O, Hochhauser D, Arnold D, Oh D-Y (2019). Maintenance olaparib for germline BRCA -mutated metastatic pancreatic cancer. N Engl J Med.

[19] Youssef ASED, Abdel-Fattah MA, Lotfy MM, Nassar A, Abouelhoda M, Touny AO, Hassan ZK, Eldin MM, Bahnassy AA, Khaled H (2022). Multigene panel sequencing reveals cancer-specific and common somatic mutations in colorectal cancer patients: an Egyptian experience. Curr Issues Mol Biol.

[20] Metwally SA, Abo-Shadi MA, Abdel Fattah NF, Barakat AB, Rabee OA, Osman AM, Helal AM, Hashem T, Moneer MM, Chehadeh W (2021). Presence of Hpv, Ebv and Hmtv Viruses among Egyptian breast cancer women: molecular detection and clinical relevance. Infect Drug Resist.

[21] Gudmundsson S, Singer-Berk M, Watts NA, Phu W, Goodrich JK, Solomonson M, Rehm HL, MacArthur DG, O’Donnell-Luria A (2022). Variant interpretation using population databases: lessons from GnomAD. Hum Mutat.

[22] Kumar P, Henikoff S, Ng PC (2009). Predicting the effects of coding non-synonymous variants on protein function using the SIFT algorithm. Nat Protoc.

[23] Landrum MJ, Lee JM, Benson M, Brown GR, Chao C, Chitipiralla S, Gu B, Hart J, Hoffman D, Jang W (2018). ClinVar: improving access to variant interpretations and supporting evidence. Nucleic Acids Res.

[24] Rentzsch P, Witten D, Cooper GM, Shendure J, Kircher M (2019). CADD: predicting the deleteriousness of variants throughout the human genome. Nucleic Acids Res.

[25] Richards S, Aziz N, Bale S, Bick D, Das S, Gastier-Foster J, Grody WW, Hegde M, Lyon E, Spector E (2015). Standards and Guidelines for the interpretation of sequence variants: a joint consensus recommendation of the American College of Medical Genetics and Genomics and the Association for Molecular Pathology. Genet Med.

[26] Oh M, McBride A, Yun S, Bhattacharjee S, Slack M, Martin JR, Jeter J, Abraham I (2018). BRCA1 and BRCA2 gene mutations and colorectal cancer risk: systematic review and meta-analysis. J Natl Cancer Inst.

[27] Kupfer SS, Gupta S, Weitzel JN, Samadder J (2020). AGA clinical practice update on colorectal and pancreatic cancer risk and screening in BRCA1 and BRCA2 carriers: commentary. Gastroenterology.

[28] Mersch J, Jackson MA, Park M, Nebgen D, Peterson SK, Singletary C, Arun BK, Litton JK (2015). Cancers associated with BRCA1 and BRCA2 mutations other than breast and ovarian. Cancer.

[29] Sopik V, Phelan C, Cybulski C, Narod SA (2015). BRCA1 and BRCA2 mutations and the risk for colorectal cancer. Clin Genet.

[30] Phelan CM, Iqbal J, Lynch HT, Lubinski J, Gronwald J, Moller P, Ghadirian P, Foulkes WD, Armel S, Eisen A (2014). Incidence of colorectal cancer in BRCA1 and BRCA2 mutation carriers: results from a follow-up study. Br J Cancer.

[31] Mavaddat N, Peock S, Frost D, Ellis S, Platte R, Fineberg E, Evans DG, Izatt L, Eeles RA, Adlard J (2013). Cancer risks for BRCA1 and BRCA2 mutation carriers: results from prospective analysis of EMBRACE. J Natl Cancer Inst.

[32] Maxwell KN, Domchek SM, Nathanson KL, Robson ME (2016). Population frequency of germline BRCA1/2 mutations. J Clin Oncol.

[33] Chen W, Xia W, Xue S, Huang H, Lin Q, Liu Y, Liu T, Zhang Y, Zhang P, Wang J (2022). Analysis of BRCA germline mutations in chinese prostate cancer patients. Front Oncol.

[34] Szczerba E, Kaminska K, Mierzwa T, Misiek M, Janusz Kowalewski MA (2021). Lewandowska BRCA1/2 mutation detection in the tumor tissue from. Genes (Basel).

[35] Darabi S, Braxton DR, Xiu J, Carneiro BA, Swensen J, Antonarakis ES, Liu SV, McKay RR, Spetzler D, El-Deiry WS, Demeure MJ (2022). BRCA1/2 reversion mutations in patients treated with poly ADP-ribose polymerase (PARP) inhibitors or platinum agents. Medicina.

[36] Analysis, D. Clinical significance of germline cancer predisposing variants in unselected patients with pancreatic adenocarcinoma. 2021. pp 1–8 10.3390/cancers13020198 PMC7827324 33429865

[37] Ademuyiwa FO, Salyer P, Ma Y, Fisher S, Colditz G, Weilbaecher K, Bierut LJ (2019). Assessing the effectiveness of the national comprehensive cancer network genetic testing guidelines in identifying african american breast cancer patients with deleterious genetic mutations. Breast Cancer Res Treat.

[38] Scandolara TB, Valle SF, Teixeira CE, Scherer NDM, de Armas EM, Furtado C, Boroni M, Jaques HDS, Alves FM, Rech D, Panis C (2022). Somatic DNA damage response and homologous repair gene alterations and its association with tumor variant burden in breast cancer patients with occupational exposure to pesticides. Front Oncol.

[39] Shi T, Wang P, Xie C, Yin S, Shi D, Wei C, Tang W, Jiang R, Cheng X, Wei Q (2017). BRCA1 and BRCA2 mutations in ovarian cancer patients from China: ethnic-related mutations in BRCA1 associated with an increased risk of ovarian cancer. Int J Cancer.

[40] Van Der Stoep N, Van Paridon CDM, Janssens T, Krenkova P, Stambergova A, Macek M, Matthijs G, Bakker E (2009). diagnostic guidelines for high-resolution melting curve (HRM) analysis: an interlaboratory validation of BRCA1 mutation scanning using the 96-well LightScanner™. Hum Mutat.

[41] Michils G, Hollants S, Dehaspe L, Van Houdt J, Bidet Y, Uhrhammer N, Bignon YJ, Vermeesch JR, Cuppens H, Matthijs G (2012). Molecular analysis of the breast cancer genes BRCA1 and BRCA2 using amplicon-based massive parallel pyrosequencing. J Mol Diagnostics.

[42] Kadri MSN, Patel KM, Bhargava PA, Shah FD, Badgujar NV, Tarapara BV, Patel PS, Shaikh MI, Shah K, Patel A (2021). Mutational landscape for indian hereditary breast and ovarian cancer cohort suggests need for identifying population specific genes and biomarkers for screening. Front Oncol.

[43] Le TNN, Tran VK, Nguyen TT, Vo NS, Hoang TH, Vo HL, Nguyen THT, Nguyen PD, Nguyen VT, Van TT (2022). BRCA1/2 mutations in vietnamese patients with hereditary breast and ovarian cancer syndrome. Genes (Basel).

[44] Wang X, Kaneko K, Arakawa H, Habano E, Omi M, Nakashima E, Kawachi H, Tonooka A, Omatsu K, Nomura H (2022). Detection of BRCA1 pathogenic variant in a 24-year-old endometrial cancer patient: risks of several hereditary tumor syndromes assessed using germline multigene panel testing. Case Rep Oncol.

[45] Eoh KJ, Kim HM, Lee JY, Kim S, Kim SW, Kim YT, Nam EJ (2020). Mutation landscape of germline and somatic BRCA1/2 in patients with high-grade serous ovarian cancer. BMC Cancer.

[46] Loza P, Irmejs A, Daneberga Z, Miklasevics E, Berga-Svitina E, Subatniece S, Maksimenko J, Trofimovics G, Tauvena E, Ukleikins S (2021). A novel frequent BRCA1 recurrent variant c.5117G > A (p.Gly1206Glu) identified after 20 years of BRCA1/2 research in the baltic region: cohort study and literature review. Hered Cancer Clin Pract.

[47] Vidal AF, Ferraz RS, El-Husny A, Silva CS, Vinasco-Sandoval T, Magalhães L, Raiol-Moraes M, Barra WF, Pereira CLBL, de Assumpção PP (2021). Comprehensive analysis of germline mutations in Northern Brazil: A panel of 16 genes for hereditary cancer-predisposing syndrome investigation. BMC Cancer.

[48] Figlioli G, De Nicolo A, Catucci I, Manoukian S, Peissel B, Azzollini J, Beltrami B, Bonanni B, Calvello M, Bondavalli D (2021). Analysis of Italian BRCA1/2 pathogenic variants identifies a private spectrum in the population from the bergamo province in Northern Italy. Cancers (Basel).

[49] Kwong A, Shin VY, Au CH, Law FBF, Ho DN, Ip BK, Wong ATC, Lau SS, To RMY, Choy G (2016). Detection of germline mutation in hereditary breast and/or ovarian cancers by next-generation sequencing on a four-gene panel. J Mol Diagnostics.

[50] Wang N, Li K, Huang W, Kong W, Liu X, Shi W, Xie F, Jiang H, Song G, Di L (2020). Efficacy of platinum in advanced triple-negative breast cancer with germline BRCA mutation determined by next generation sequencing. Chin J Cancer Res.

[51] Chan GHJ, Ong PY, Low JJH, Kong HL, Ow SGW, Tan DSP, Lim YW, Lim SE, Lee SC (2018). Clinical genetic testing outcome with multi-gene panel in Asian patients with multiple primary cancers. Oncotarget.

[52] Lertwilaiwittaya P, Roothumnong E, Nakthong P, Dungort P, Meesamarnpong C, Tansa-Nga W, Pongsuktavorn K, Wiboonthanasarn S, Tititumjariya W, Thongnoppakhun W (2021). Thai patients who fulfilled NCCN criteria for breast/ovarian cancer genetic assessment demonstrated high prevalence of germline mutations in cancer susceptibility genes: implication to Asian population testing. Breast Cancer Res Treat.

[53] Choi MC, Hwang S, Kim S, Jung SG, Park H, Joo WD, Song SH, Lee C, Kim TH, Kang H (2020). Clinical impact of somatic variants in homologous recombination repair-related genes in ovarian high-grade serous carcinoma. Cancer Res Treat.

[54] Wu H, Zhou L, Zhou X, Wei Q, Ouyang N, Shao J, Huang J, Liang Z (2022). Challenges in next generation sequencing of homology recombination repair genomic variants in prostate cancer: a nationwide survey and calibration project in China. Prostate Int.

[55] Koczkowska M, Zuk M, Gorczynski A, Ratajska M, Lewandowska M, Biernat W, Limon J, Wasag B (2016). Detection of somatic BRCA1/2 mutations in ovarian cancer—next-generation sequencing analysis of 100 cases. Cancer Med.

[56] Felix GES, Guindalini RSC, Zheng Y, Walsh T, Sveen E, Lopes TMM, Côrtes J, Zhang J, Carôzo P, Santos I (2022). Mutational spectrum of breast cancer susceptibility genes among women ascertained in a cancer risk clinic in northeast Brazil. Breast Cancer Res Treat.

[57] Li Y, Chen L, Lv J, Chen X, Zeng B, Chen M, Guo W, Lin Y, Yu L, Hou J (2022). Clinical application of artificial neural network (ANN) modeling to predict BRCA1/2 germline deleterious variants in Chinese bilateral primary breast cancer patients. BMC Cancer.

[58] Bisgin A, Sag SO, Dogan ME, Yildirim MS, Gumus AA, Akkus N, Balasar O, Durmaz CD, Ersoz R, Altiner S (2022). Germline landscape of BRCAs by 7-site collaborations as a BRCA consortium in Turkey. Breast.

[59] Schenkel LC, Kerkhof J, Stuart A, Reilly J, Eng B, Woodside C, Levstik A, Howlett CJ, Rupar AC, Knoll JHM (2016). Clinical next-generation sequencing pipeline outperforms a combined approach using sanger sequencing and multiplex ligation-dependent probe amplification in targeted gene panel analysis. J Mol Diagn.

[60] Alunni-fabbroni M, Weber S, Öcal O, Seidensticker M, Mayerle J, Malfertheiner P, Ricke J (2021). Circulating cell-free dna combined to magnetic resonance imaging for early detection of hcc in patients with liver cirrhosis. Cancers (Basel).

[61] Paik ES, Heo EJ, Choi CH, Kim JH, Kim JW, Kim YM, Park SY, Lee JW, Kim JW, Kim BG (2021). Prevalence and clinical characterization of BRCA1 and BRCA2 mutations in korean patients with epithelial ovarian cancer. Cancer Sci.

[62] Li G, Guo X, Tang L, Chen M, Luo X, Peng L, Xu X, Wang S, Xiao Z, Yi W (2017). Analysis of BRCA1/2 mutation spectrum and prevalence in unselected chinese breast cancer patients by next-generation sequencing. J Cancer Res Clin Oncol.

[63] Solano AR, Cardoso FC, Romano V, Perazzo F, Bas C, Recondo G, Santillan FB, Gonzalez E, Abalo E, Viniegra M (2017). Spectrum of BRCA1/2 variants in 940 patients from Argentina including novel, deleterious and recurrent germline mutations: impact on healthcare and clinical practice. Oncotarget.

[64] Vidra R, Ciuleanu TE, Nemeș A, Pascu O, Heroiu AM, Antone N, Vidrean AI, Oprean CM, Pop LA, Berindan-Neagoe I (2022). Article spectrum of BRCA1/2 mutations in Romanian breast and ovarian cancer patients. Int J Environ Res Public Health.

[65] Bhaskaran SP, Chandratre K, Gupta H, Zhang L, Wang X, Cui J, Kim YC, Sinha S, Jiang L, Lu B (2019). Germline variation in BRCA1/2 is highly ethnic-specific: evidence from over 30,000 chinese hereditary breast and ovarian cancer patients. Int J Cancer.

[66] Laish I, Friedman E, Levi-Reznick G, Kedar I, Katz L, Levi Z, Halpern N, Parnasa S, Abu-Shatya A, Half E (2021). Double heterozygotes of BRCA1/BRCA2 and mismatch repair gene pathogenic variants: case series and clinical implications. Breast Cancer Res Treat.

[67] Saied MH, Elkaffash D, Fadl R, Haleem RA, Refeat A, Ibrahim I, Tahoun M, Elkayal A, Tayae E (2021). Preliminary results of targeted sequencing of BRCA1 and BRCA2 in a cohort of breast cancer families: new insight into pathogenic variants in patients and at-risk relatives. Mol Med Rep.

[68] Cherbal F, Salhi N, Bakour R, Adane S, Boualga K, Maillet P (2012). BRCA1 and BRCA2 unclassified variants and missense polymorphisms in algerian breast/ovarian cancer families. Dis Markers.

[69] Al Hannan F, Keogh MB, Taha S, Al Buainain L (2019). Characterization of BRCA1 and BRCA2 genetic variants in a cohort of Bahraini breast cancer patients using next-generation sequencing. Mol Genet Genomic Med.

[70] Salmi F, Maachi F, Tazzite A, Aboutaib R, Fekkak J, Azeddoug H, Jouhadi H (2021). Next-generation sequencing of BRCA1 and BRCA2 genes in Moroccan prostate cancer patients with positive family history. PLoS ONE.

[71] Ben Ayed-Guerfali D, Ben Kridis-Rejab W, Ammous-Boukhris N, Ayadi W, Charfi S, Khanfir A, Sellami-Boudawara T, Frikha M, Daoud J, Mokdad-Gargouri R (2021). Novel and recurrent BRCA1/BRCA2 germline mutations in patients with breast/ovarian cancer: a series from the South of Tunisia. J Transl Med.

[72] Miao Y, Tang S (2022). Detection of breast cancer lump and BRCA1/2 genetic mutation under deep learning. Comput Intell Neurosci.

[73] Kim JH, Park S, Park HS, Park JS, Lee ST, Kim SW, Lee JW, Lee MH, Park SK, Noh WC (2021). Analysis of BRCA1/2 variants of unknown significance in the prospective Korean hereditary breast cancer study. Sci Rep.

[74] Forat-Yazdi M, Neamatzadeh H, Sheikhha MH, Zare-Shehneh M, Fattahi M (2015). BRCA1 and BRCA2 common mutations in iranian breast cancer patients: a meta analysis. Asian Pacific J Cancer Prev.

[75] Shah ND, Shah PS, Panchal YY, Katudia KH, Khatri NB, Ray HSP, Bhatiya UR, Shah SC, Shah BS, Rao MV (2018). Mutation analysis of BRCA1/2 mutations with special reference to polymorphic SNPs in Indian breast cancer patients. Appl Clin Genet.

[76] Da Costa E, Silva Carvalho S, Cury NM, Brotto DB, De Araujo LF, Rosa RCA, Texeira LA, Plaça JR, Marques AA, Peronni KC, Ruy PDC (2020). Germline variants in DNA repair genes associated with hereditary breast and ovarian cancer syndrome: analysis of a 21 gene panel in the Brazilian population. BMC Med Genomics.

[77] T&D Electrical Risk assessment risk assessment risk assessment. Risk Manag. 2008, 24, 1–7

[78] Ibragimova MK, Tsyganov MM, Litviakov NV (2018). Human papillomavirus and colorectal cancer. Med Oncol.

[79] Ojesina AI, Lichtenstein L, Freeman SS, Pedamallu CS, Imaz-Rosshandler I, Pugh TJ, Cherniack AD, Ambrogio L, Cibulskis K, Bertelsen B (2014). Landscape of genomic alterations in cervical carcinomas. Nature.

[80] Khoury JD, Tannir NM, Williams MD, Chen Y, Yao H, Zhang J, Thompson EJ, Meric-Bernstam F, Medeiros LJ, Weinstein JN (2013). Landscape of DNA virus associations across human malignant cancers: analysis of 3,775 cases using RNA-Seq. J Virol.

