# Supplementary material for: Deleterious and ethnic-related BRCA1/2 mutations in tissue and blood of Egyptian colorectal cancer patients and its correlation with human papillomavirus
Source: Clin Exp Med. 2023 Oct 7;23(8):5063–88. doi: 10.1007/s10238-023-01207-w (PMC10725364; doi:10.1007/s10238-023-01207-w)
Supplement: Supplementary file 1 — Supplementary file1 (DOCX 5676 KB) [file 10238_2023_1207_MOESM1_ESM.docx]

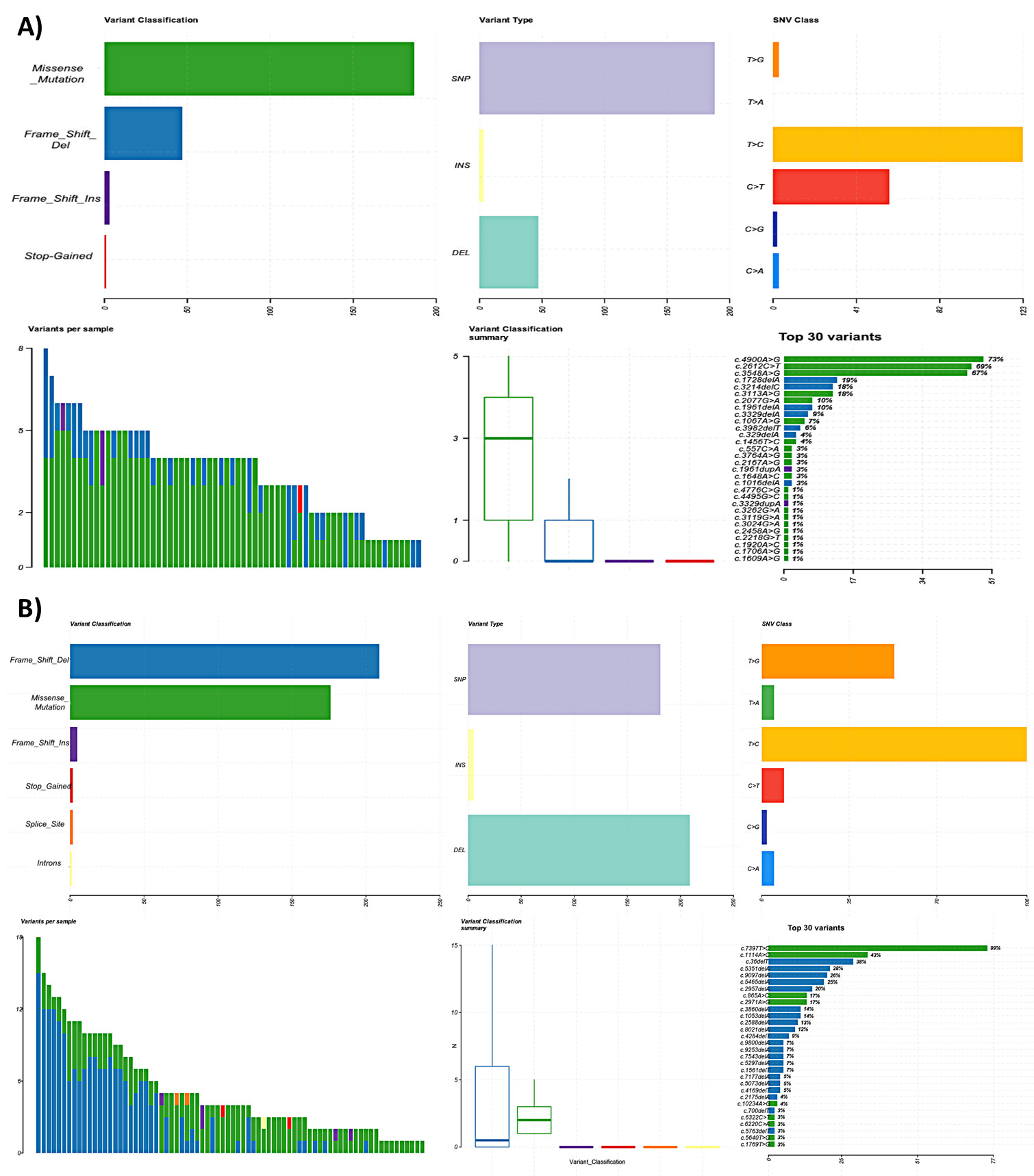


**Figure S1:** Summary of (**A**) *BRCA1,* and (**B**) *BRCA2* mutations detected in the tissue of the CRC patients.


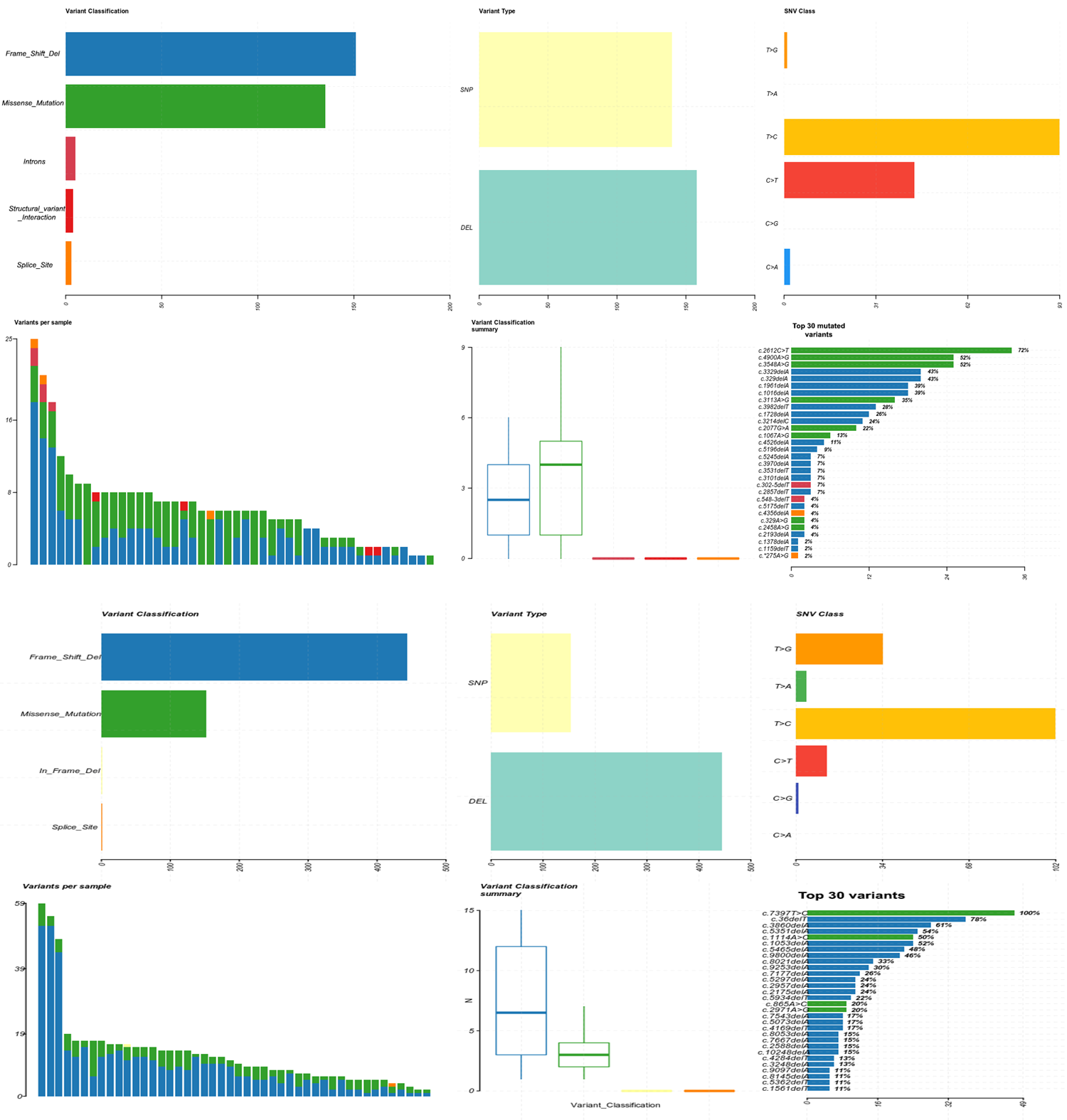


**Figure S2:** Pie charts show *BRCA1/2* (**A**) variants classification, and (**B**) clinical significance in the blood of the CRC patients.


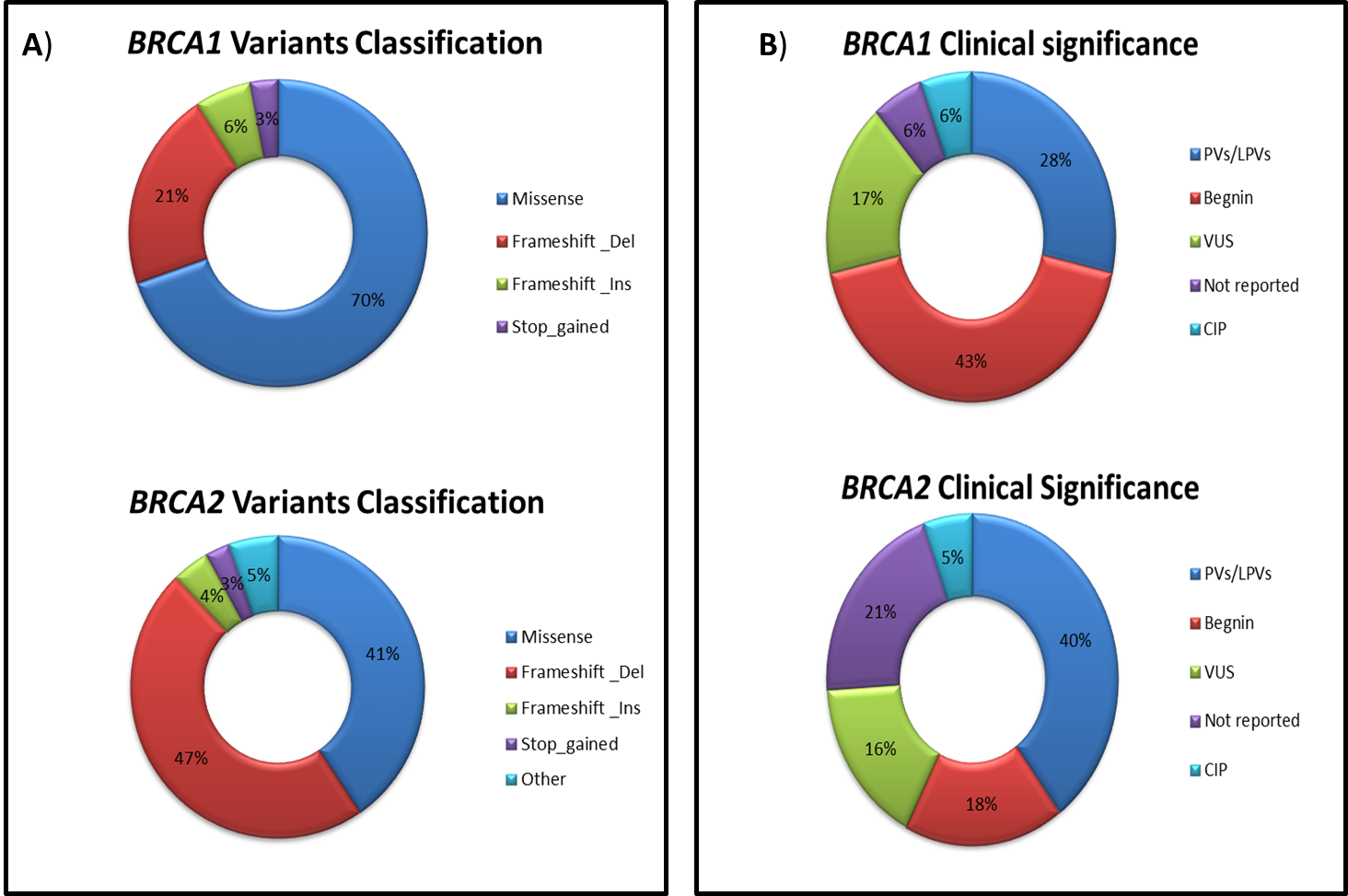


**Figure S3:** Summary of (**A**) *BRCA1,* and (**B**) *BRCA2* mutations detected in the tissue of the CRC patients.


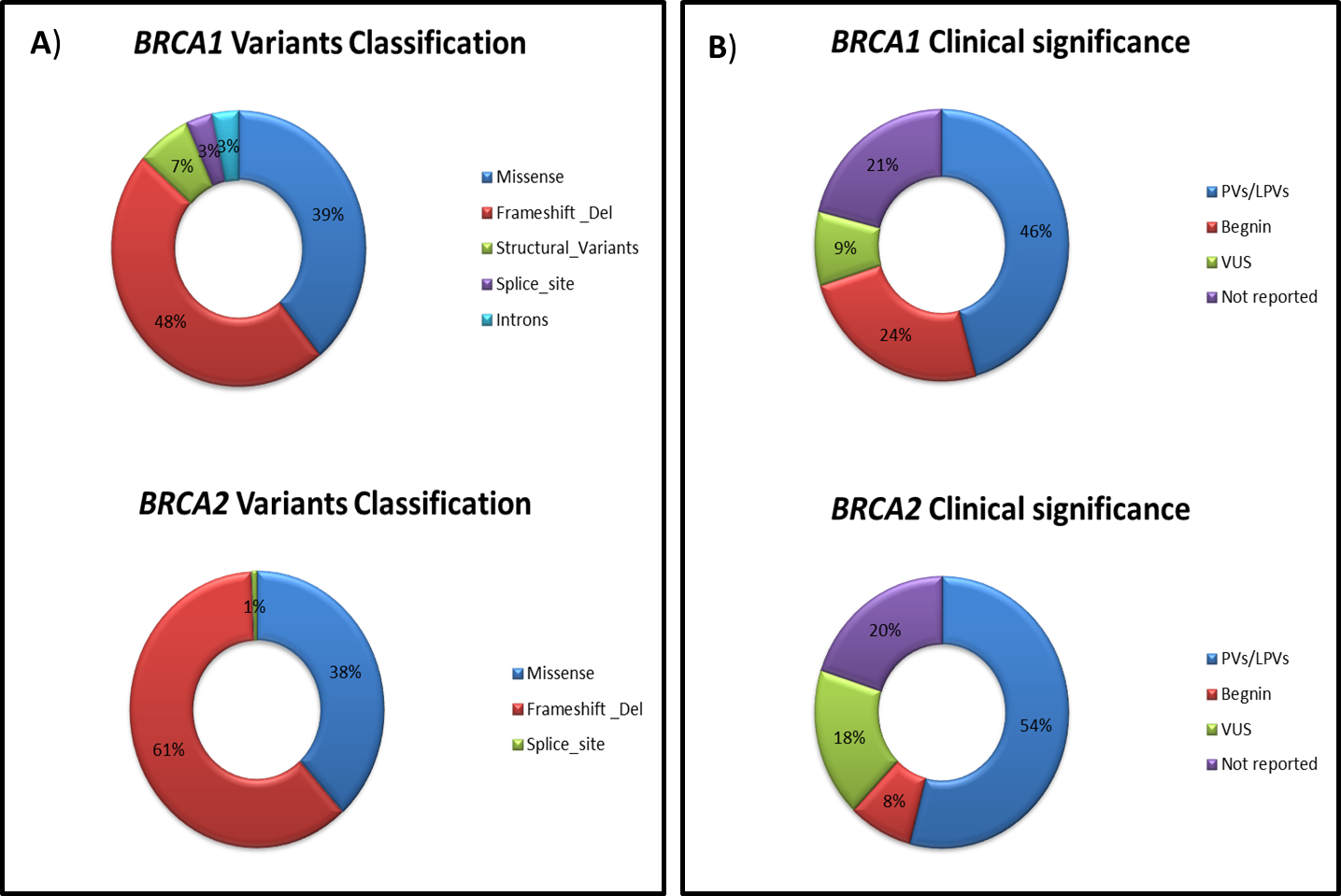


**Figure S4:** Pie charts show *BRCA1/2* (**A**) variants classification, and (**B**) clinical significance in the blood of the CRC patients.


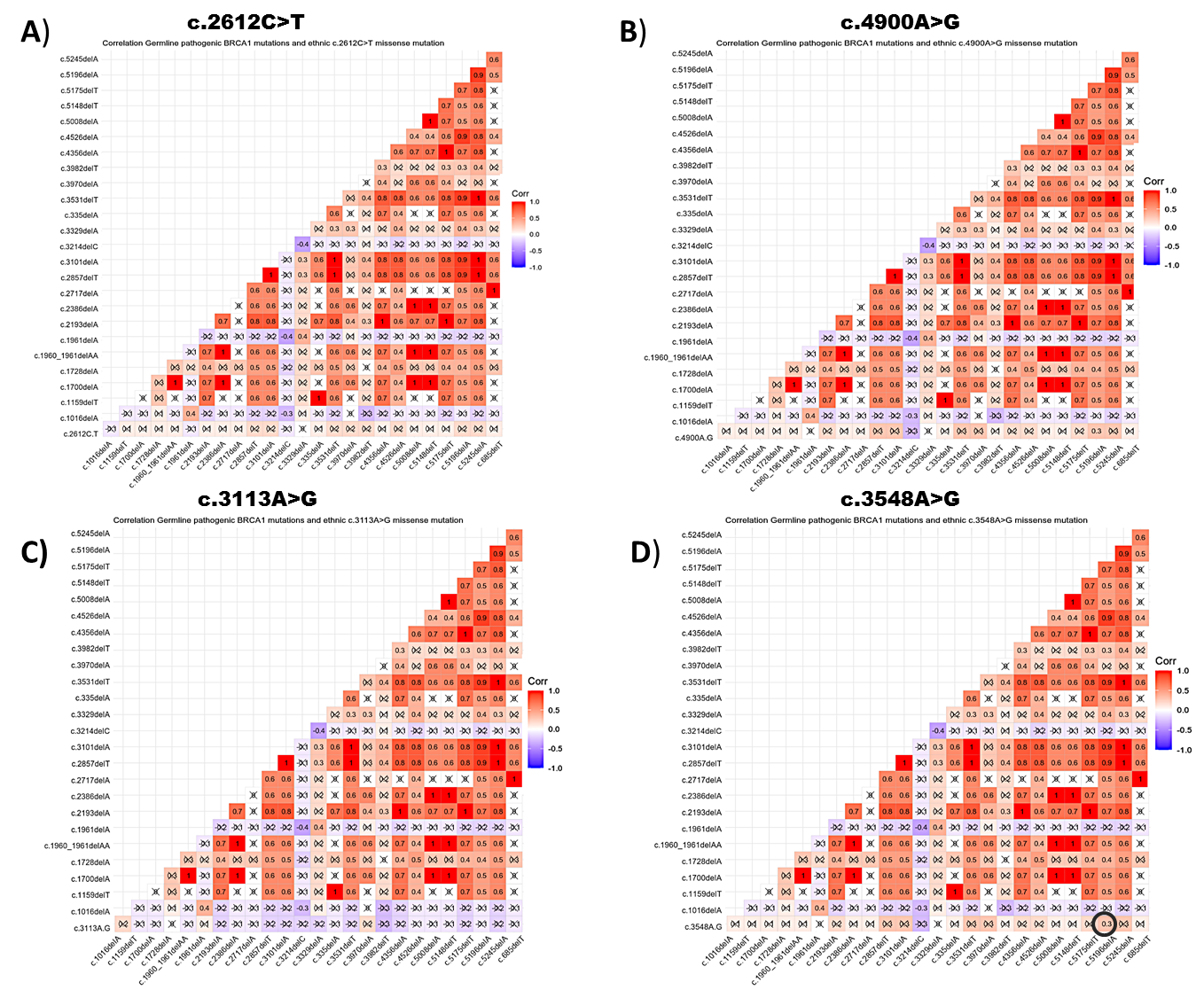


**Figure S5:** Correlation matrix between ethnic-related and pathogenic *BRCA1* variants in the blood of the CRC patients.


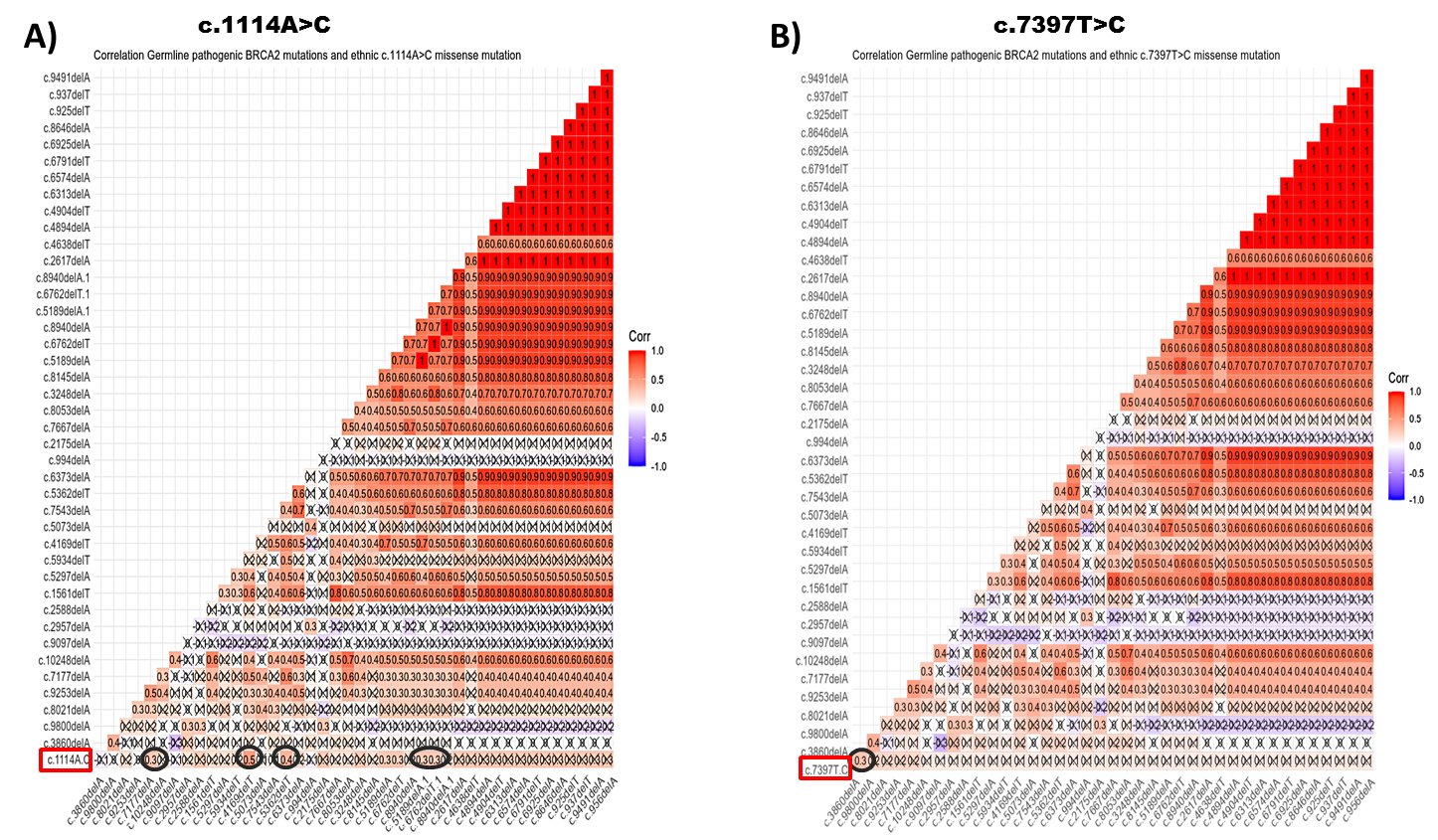


**Figure S6:** Correlation matrix between ethnic-related and pathogenic *BRCA2* variants in the blood of the CRC patients.

**Table S1:** Clinicopathological features of the CRC group.

| **Age (mean ±SD), Total=82** | 50.48 ±12.09 |
| --- | --- |
| **Gender (n (%))** |  |
| Male | 43 (52.43) |
| Female | 39 (47.57) |
| **Site (n (%))** |  |
| Colon | 29 (35.37) |
| Rectum | 41 (50.0) |
| Cecum | 2 (2.44) |
| Sigmoid | 10 (12.19) |
| **Type (n (%))** |  |
| Adenocarcinoma | 69 (84.14) |
| Mucinous adenocarcinoma | 4 (4.88) |
| Signet ring adenocarcinoma | 4 (4.88) |
| Neuroendocrine | 3 (3.66) |
| Squamous cell carcinoma | 1 (1.22) |
| B-cell NHL and Burkett’s lymphoma | 1 (1.22) |
| **Grade (n (%))** |  |
| 1 | 14 (17.07) |
| 2 | 49 (59.76) |
| 3 | 19 (23.17) |
| **Recurrence (n (%))** |  |
| Yes | 4 (4.88) |
| No | 78 (95.12) |
| **Metastasis (n (%))** |  |
| Yes | 4 (4.88) |
| No | 78 (95.12) |
| **History of any cancer (n (%))** |  |
| Yes | 4 (4.88) |
| No | 78 (95.12) |
| **HPV (n (%)), Total=63** |  |
| Negative | 15 (23.8) |
| Positive | 48 (76.2) |

**Table S2:** Shared *BRCA1/2* pathogenic mutations detected in the blood of the CRC patients compared to the blood of healthy controls.

| ***Gene*** | **Position** | **Exon** | **ID** | **Clinical**  **Significance** | **HGVS.c** | **CRC Blood**  **(n=46)** | **Control Blood**  **(n=43)** |  | **OR (95% CI)** | **P-value** |
| --- | --- | --- | --- | --- | --- | --- | --- | --- | --- | --- |
| ***BRCA1*** | Chr17:41256250 | 6 | COSM1383528  ;rs80357604 | PV | c.329delA | **20 (43%)** | **10 (23%)** |  | **2.5 (1.2 to 6.3)** | **0.052** |
|  | Chr17: 41244218 | 10 | rs80357575 | PV | c.3329delA | **20 (43%)** | **7 (16%)** |  | **4 (1.2 to 11.3)** | **0.0069**** |
|  | Chr17: 41245586 | 10 | COSM219054  ;rs80357522 | PV | c.1961delA | **18 (39%)** | **9 (21%)** |  | **2.8 (1.1to 7.4)** | **0.06** |
|  | Chr17: 41246531 | 10 | rs80357569 | PV | c.1016delA | **18 (39%)** | **6 (14%)** |  | **3.5 (1.4 to 11.2)** | **0.0099**** |
|  | Chr17: 41243565 | 10 | SCV001499582.1 | NPV | c.3982delT | **13 (28%)** | 8 (18%) |  | 1.7 ( 0.63 to 4.68) | 0.28 |
|  | Chr17: 41245819 | 10 | rs397507192 | PV | c.1728delA | 12 (26%) | 7(16%) |  | 1.8 (0.64 to 5.2) | 0.26 |
|  | Chr17: 41244333 | 10 | rs80357923 | PV | c.3214delC | 11 (24%) | 9 (21%) |  | 1.18 (0.43 to 3.2) | 0.73 |
| ***BRCA2*** | Chr13: 32907171 | 10 | rs886040374 | NPV | c.1561delT | 5 (11%) | 2 (5%) |  | 2.5 ( 0.46 to 13.6) | 0.29 |
|  | Chr13: 32906602 | 10 | rs80359777 | PV | c.994delA | 5 (11%) | **0 (0%)** |  | 11.5 ( 0.6180 to 215) | 0.10 |
|  | Chr13: 32906888 | 10 | rs80359274 | PV | c.1278delA | 2 (4%) | 0 (0%) |  | 4.8 ( 0.2280 to 105) | 0.31 |
|  | Chr13: 32912345 | 11 | rs80359406 | PV | c.3860delA | **28 (61%)** | **9 (21%)** |  | **5.8 ( 2.3 to 15.1)** | **0.0002**** |
|  | Chr13: 32910661 | 11 | rs276174819 | PV | c.2175delA | 11 (24%) | **3 (7%)** |  | **4.2 (1.1 to 16.2)** | **0.038*** |
|  | Chr13: 32913783 | 11 | rs1555284157 | PV | c.5297delA | 11 (24%) | **3 (7%)** |  | **4.2 (1.1 to 16.2)** | **0.038*** |
|  | Chr13: 32912655 | 11 | rs80359433 | PV | c.4169delT | 8 (17%) | **1 (2%)** |  | **8.8 (1.1 to 74)** | **0.044*** |
|  | Chr13: 32913558 | 11 | COSM1562291;  rs80359479 | PV | c.5073delA | 8 (17%) | **2 (5%)** |  | **8.8 (1.1 to 74)** | **0.07** |
|  | Chr13: 32914859 | 11 | rs80359578 | PV | c.6373delA | 4 (9%) | 0 (0%) |  | 9.2 (0.48 to 176) | 0.14 |
|  | Chr13: 32929161 | 14 | rs397507899 | NPV | c.7177delA | 12 (26%) | **3 (7%)** |  | **4.7 (1.2 to 18)** | **0.02*** |
|  | Chr13: 32930667 | 15 | rs80359657 | PV | c.7543delA | 8 (17%) | 2 (5%) |  | 4.3 (0.86 to 21.6) | 0.07 |
|  | Chr13: 32937354 | 18 | rs397507952 | PV | c.8021delA | **15 (33%)** | **7 (16%)** |  | **2.9 (1.1 to 8.6)** | **0.072** |
|  | Chr13: 32954022 | 23 | COSM1366492  ; rs397507419 | PV | c.9097delA | 5 (11%) | 7 (16%) |  | 0.62 (0.18 to 2.1) | 0.45 |
|  | Chr13: 32954272 | 24 | rs80359752 | PV | c.9253delA | **14 (30%)** | **4 (9%)** |  | **4.3 (1.3 to 14.2)** | **0.01**** |
|  | Chr13: 32972445 | 27 | rs1566261027 | NPV | c.9800delA | **21 (46%)** | **10 (23%)** |  | **2.7 (1.1 to 6.9)** | **0.029*** |
|  | Chr13: 32972892 | 27 | COSM309515  ;rs776212316 | NPV | c.10248delA | 7 (11%) | 3 (7%) |  | 2.4 (0.57 to 9.9) | 0.22 |

**HGVS.c**: Human Genome Variation Society, coding DNA sequence; **HGVS.p:** Human Genome Variation Society, protein sequence; **Chr.**: Chromosome; **CRC**: Colorectal Cancer; **PV**: Pathogenic Variants; **NPV**: Novel Pathogenic Variant; **VMF**: Variant Major Allele Frequency; **Del**: Deletion; **OR:** Odds Ratio.

**Table S3:** Co-occurrence and mutually exclusive events of *BRCA1/2* pathogenic mutations in CRC tissue.

| **BRCA2** | **BRCA1** | **pValue** | **oddsRatio** | **0** | **11** | **1** | **10** | **Event** | **pair** | **event_ratio** |
| --- | --- | --- | --- | --- | --- | --- | --- | --- | --- | --- |
| c.3329delA | c.1728delA | 0.004235035 | 18.92013905 | 33 | 5 | 8 | 1 | Co_Occurence | c.1728delA, c.3329delA | 5/9 |
| c.5297delA | c.1561delT | 0.005750555 | 25.07055892 | 40 | 3 | 2 | 2 | Co_Occurence | c.1561delT, c.5297delA | 3/4 |
| c.9097delA | c.1728delA | 0.006899355 | 7.605921268 | 24 | 10 | 3 | 10 | Co_Occurence | c.9097delA, c.1728delA | 10/13 |
| c.4284delT | c.4169delT | 0.0080453 | 25.23683491 | 39 | 3 | 1 | 4 | Co_Occurence | c.4169delT, c.4284delT | 3/5 |
| c.5073delA | c.1961delA | 0.0080453 | 25.23683491 | 39 | 3 | 4 | 1 | Co_Occurence | c.5073delA, c.1961delA | 3/5 |
| c.9097delA | c.9800delA | 0.010107312 | Inf | 27 | 5 | 0 | 15 | Co_Occurence | c.9097delA, c.9800delA | 5/15 |
| c.9800delA | c.1728delA | 0.016687104 | 13.62727812 | 33 | 4 | 9 | 1 | Co_Occurence | c.1728delA, c.9800delA | 4/10 |
| c.2588delA | c.1728delA | 0.017496175 | 6.109556081 | 30 | 6 | 7 | 4 | Co_Occurence | c.1728delA, c.2588delA | 6/11 |
| c.8021delA | c.4284delT | 0.01836086 | 8.67193018 | 35 | 4 | 3 | 5 | Co_Occurence | c.4284delT, c.8021delA | 4/8 |
| c.4284delT | c.1561delT | 0.018723691 | 12.8307101 | 38 | 3 | 2 | 4 | Co_Occurence | c.1561delT, c.4284delT | 3/6 |
| c.9800delA | c.1961delA | 0.018723691 | 12.8307101 | 38 | 3 | 4 | 2 | Co_Occurence | c.9800delA, c.1961delA | 3/6 |
| c.4284delT | c.9800delA | 0.018723691 | 12.8307101 | 38 | 3 | 2 | 4 | Co_Occurence | c.4284delT, c.9800delA | 3/6 |
| c.7177delA | c.2588delA | 0.026070137 | 14.13456923 | 36 | 3 | 7 | 1 | Co_Occurence | c.2588delA, c.7177delA | 3/8 |
| c.2957delA | c.1961delA | 0.026332382 | 7.12195251 | 30 | 5 | 2 | 10 | Co_Occurence | c.1961delA, c.2957delA | 5/12 |
| c.2957delA | c.4284delT | 0.026332382 | 7.12195251 | 30 | 5 | 2 | 10 | Co_Occurence | c.2957delA, c.4284delT | 5/12 |
| c.9097delA | c.2957delA | 0.029921398 | 4.249736154 | 22 | 10 | 5 | 10 | Co_Occurence | c.2957delA, c.9097delA | 10/15 |
| c.1561delT | c.2957delA | 0.030433414 | 10.62015314 | 31 | 4 | 11 | 1 | Co_Occurence | c.1561delT, c.2957delA | 4/12 |
| c.5297delA | c.2957delA | 0.030433414 | 10.62015314 | 31 | 4 | 11 | 1 | Co_Occurence | c.2957delA, c.5297delA | 4/12 |
| c.9800delA | c.2957delA | 0.030433414 | 10.62015314 | 31 | 4 | 11 | 1 | Co_Occurence | c.2957delA, c.9800delA | 4/12 |
| c.9097delA | c.4284delT | 0.031992718 | 10.59617359 | 26 | 6 | 1 | 14 | Co_Occurence | c.4284delT, c.9097delA | 6/15 |
| c.1561delT | c.8021delA | 0.041700485 | 8.374115857 | 36 | 3 | 6 | 2 | Co_Occurence | c.1561delT, c.8021delA | 3/8 |
| c.7543delA | c.8021delA | 0.041700485 | 8.374115857 | 36 | 3 | 6 | 2 | Co_Occurence | c.7543delA, c.8021delA | 3/8 |
| c.1961dupA | c.3329dupA | 0.042553191 | Inf | 45 | 1 | 0 | 1 | Co_Occurence | c.1961dupA, c.3329dupA | 1/1 |
| c.9097dupA | c.5566_5567delCAinsTG | 0.042553191 | Inf | 45 | 1 | 0 | 1 | Co_Occurence | c.5566_5567delCAinsTG,  c.9097dupA | 1/1 |
| c.700delT | c.6373delA | 0.042553191 | Inf | 45 | 1 | 0 | 1 | Co_Occurence | c.6373delA, c.700delT | 1/1 |

**Table S4:** Co-occurrence and mutually exclusive events of *BRCA1/2* pathogenic mutations in CRC blood.

| **BRCA2** | **BRCA1** | **pValue** | **oddsRatio** | **0_0** | **11** | **0_1** | **10** | **Event** | **pair** | **event_ratio** |
| --- | --- | --- | --- | --- | --- | --- | --- | --- | --- | --- |
| c.9800delA | c.1961delA | 3.43606E-06 | 31.24532701 | 22 | 16 | 2 | 5 | Co_Occurence | c.1961delA, c.9800delA | 16/7 |
| c.9253delA | c.1728delA | 1.658E-05 | 31.61816212 | 29 | 10 | 2 | 4 | Co_Occurence | c.9253delA, c.1728delA | 10/6 |
| c.7667delA | c.1561delT | 1.71883E-05 | Inf | 38 | 5 | 0 | 2 | Co_Occurence | c.1561delT, c.7667delA | 5/2 |
| c.3860delA | c.3214delC | 5.48657E-05 | 0.028836813 | 7 | 1 | 10 | 27 | Mutually_Exclusive | c.3214delC, c.3860delA | 1/37 |
| c.9800delA | c.3214delC | 0.000280659 | 0 | 13 | 0 | 11 | 21 | Mutually_Exclusive | c.3214delC, c.9800delA | 0/32 |
| c.8053delA | c.10248delA | 0.000331206 | 36.81657187 | 36 | 5 | 2 | 2 | Co_Occurence | c.10248delA, c.8053delA | 5/4 |
| c.7177delA | c.8053delA | 0.000689384 | 28.50063144 | 32 | 6 | 1 | 6 | Co_Occurence | c.7177delA, c.8053delA | 6/7 |
| c.9800delA | c.1016delA | 0.000881781 | 9.392289466 | 20 | 14 | 4 | 7 | Co_Occurence | c.9800delA, c.1016delA | 14/11 |
| c.9800delA | c.3329delA | 0.000954183 | 8.934065811 | 19 | 15 | 5 | 6 | Co_Occurence | c.3329delA, c.9800delA | 15/11 |
| c.1561delT | c.10248delA | 0.001105783 | 40.43218273 | 37 | 4 | 3 | 1 | Co_Occurence | c.10248delA, c.1561delT | 4/4 |
| c.1561delT | c.8053delA | 0.001105783 | 40.43218273 | 37 | 4 | 3 | 1 | Co_Occurence | c.1561delT, c.8053delA | 4/4 |
| c.3214delC | c.1961delA | 0.001403975 | 0 | 16 | 0 | 18 | 11 | Mutually_Exclusive | c.1961delA, c.3214delC | 0/29 |
| c.7667delA | c.3329delA | 0.001708256 | Inf | 25 | 7 | 13 | 0 | Co_Occurence | c.3329delA, c.7667delA | 7/13 |
| c.3248delA | c.5297delA | 0.001985252 | 24.56257129 | 33 | 5 | 6 | 1 | Co_Occurence | c.3248delA, c.5297delA | 5/7 |
| c.7543delA | c.4169delT | 0.00210651 | 16.78868825 | 34 | 5 | 3 | 3 | Co_Occurence | c.4169delT, c.7543delA | 5/6 |
| c.1561delT | c.4169delT | 0.00216573 | 30.80613436 | 36 | 4 | 4 | 1 | Co_Occurence | c.1561delT, c.4169delT | 4/5 |
| c.4169delT | c.7177delA | 0.002386896 | 14.17298819 | 31 | 6 | 6 | 2 | Co_Occurence | c.4169delT, c.7177delA | 6/8 |
| c.9253delA | c.7177delA | 0.003614922 | 8.441542993 | 27 | 8 | 4 | 6 | Co_Occurence | c.7177delA, c.9253delA | 8/10 |
| c.5073delA | c.1961delA | 0.004189249 | 15.4872739 | 26 | 7 | 11 | 1 | Co_Occurence | c.5073delA, c.1961delA | 7/12 |
| c.1961delA | c.1016delA | 0.004892116 | 6.649939296 | 21 | 12 | 6 | 6 | Co_Occurence | c.1016delA, c.1961delA | 12/12 |
| c.3860delA | c.3329delA | 0.006181838 | 6.882032948 | 14 | 17 | 3 | 11 | Co_Occurence | c.3860delA, c.3329delA | 17/14 |
| c.7667delA | c.10248delA | 0.006837651 | 13.92857754 | 35 | 4 | 3 | 3 | Co_Occurence | c.10248delA, c.7667delA | 4/6 |
| c.8053delA | c.7667delA | 0.006837651 | 13.92857754 | 35 | 4 | 3 | 3 | Co_Occurence | c.7667delA, c.8053delA | 4/6 |
| c.7543delA | c.9253delA | 0.0069857 | 10.16074487 | 29 | 6 | 8 | 2 | Co_Occurence | c.7543delA, c.9253delA | 6/10 |
| c.9253delA | c.1961delA | 0.007484186 | 6.824187713 | 23 | 10 | 8 | 4 | Co_Occurence | c.1961delA, c.9253delA | 10/12 |
| c.4284delT | c.9253delA | 0.007988278 | 15.44033652 | 30 | 5 | 9 | 1 | Co_Occurence | c.4284delT, c.9253delA | 5/10 |
| c.9253delA | c.3214delC | 0.009766237 | 0 | 20 | 0 | 11 | 14 | Mutually_Exclusive | c.3214delC, c.9253delA | 0/25 |
| c.10248delA | c.1728delA | 0.009904446 | 10.28285998 | 31 | 5 | 7 | 2 | Co_Occurence | c.10248delA, c.1728delA | 5/9 |
| c.5073delA | c.8021delA | 0.011025863 | 8.784809253 | 28 | 6 | 9 | 2 | Co_Occurence | c.5073delA, c.8021delA | 6/11 |
| c.3329delA | c.3214delC | 0.012315283 | 0.082948764 | 15 | 1 | 10 | 19 | Mutually_Exclusive | c.3214delC, c.3329delA | 1/29 |
| c.1561delT | c.3248delA | 0.012613781 | 16.19988853 | 37 | 3 | 3 | 2 | Co_Occurence | c.1561delT, c.3248delA | 3/5 |
| c.1561delT | c.3329delA | 0.0126899 | Inf | 25 | 5 | 15 | 0 | Co_Occurence | c.1561delT, c.3329delA | 5/15 |
| c.10248delA | c.4169delT | 0.012830429 | 10.37514807 | 34 | 4 | 4 | 3 | Co_Occurence | c.10248delA, c.4169delT | 4/7 |
| c.7667delA | c.4169delT | 0.012830429 | 10.37514807 | 34 | 4 | 4 | 3 | Co_Occurence | c.4169delT, c.7667delA | 4/7 |
| c.8053delA | c.4169delT | 0.012830429 | 10.37514807 | 34 | 4 | 4 | 3 | Co_Occurence | c.4169delT, c.8053delA | 4/7 |
| c.10248delA | c.7543delA | 0.012830429 | 10.37514807 | 34 | 4 | 4 | 3 | Co_Occurence | c.10248delA, c.7543delA | 4/7 |
| c.7667delA | c.7543delA | 0.012830429 | 10.37514807 | 34 | 4 | 4 | 3 | Co_Occurence | c.7543delA, c.7667delA | 4/7 |
| c.8053delA | c.7543delA | 0.012830429 | 10.37514807 | 34 | 4 | 4 | 3 | Co_Occurence | c.7543delA, c.8053delA | 4/7 |
| c.1561delT | c.1728delA | 0.014018313 | 14.73602054 | 32 | 4 | 8 | 1 | Co_Occurence | c.1561delT, c.1728delA | 4/9 |
| c.1561delT | c.7177delA | 0.014018313 | 14.73602054 | 32 | 4 | 8 | 1 | Co_Occurence | c.1561delT, c.7177delA | 4/9 |
| c.4169delT | c.5297delA | 0.014080863 | 8.052229236 | 31 | 5 | 6 | 3 | Co_Occurence | c.4169delT, c.5297delA | 5/9 |
| c.7543delA | c.5297delA | 0.014080863 | 8.052229236 | 31 | 5 | 6 | 3 | Co_Occurence | c.5297delA, c.7543delA | 5/9 |
| c.7543delA | c.3329delA | 0.014592894 | 12.22338861 | 24 | 7 | 13 | 1 | Co_Occurence | c.3329delA, c.7543delA | 7/14 |
| c.10248delA | c.3982delT | 0.015314804 | 8.78992587 | 30 | 5 | 8 | 2 | Co_Occurence | c.10248delA, c.3982delT | 5/10 |
| c.7667delA | c.3982delT | 0.015314804 | 8.78992587 | 30 | 5 | 8 | 2 | Co_Occurence | c.7667delA, c.3982delT | 5/10 |
| c.2175delA | c.1016delA | 0.015694542 | 6.107866089 | 24 | 8 | 10 | 3 | Co_Occurence | c.1016delA, c.2175delA | 8/13 |
| c.1561delT | c.3982delT | 0.019780497 | 12.80741685 | 31 | 4 | 9 | 1 | Co_Occurence | c.1561delT, c.3982delT | 4/10 |
| c.4169delT | c.1728delA | 0.022433697 | 6.742452579 | 30 | 5 | 7 | 3 | Co_Occurence | c.4169delT, c.1728delA | 5/10 |
| c.5073delA | c.1728delA | 0.022433697 | 6.742452579 | 30 | 5 | 7 | 3 | Co_Occurence | c.5073delA, c.1728delA | 5/10 |
| c.7177delA | c.5073delA | 0.022433697 | 6.742452579 | 30 | 5 | 3 | 7 | Co_Occurence | c.5073delA, c.7177delA | 5/10 |
| c.7543delA | c.1728delA | 0.022433697 | 6.742452579 | 30 | 5 | 7 | 3 | Co_Occurence | c.7543delA, c.1728delA | 5/10 |
| c.10248delA | c.9253delA | 0.022641331 | 7.607465547 | 29 | 5 | 9 | 2 | Co_Occurence | c.10248delA, c.9253delA | 5/11 |
| c.8053delA | c.9253delA | 0.022641331 | 7.607465547 | 29 | 5 | 9 | 2 | Co_Occurence | c.8053delA, c.9253delA | 5/11 |
| c.4284delT | c.2957delA | 0.024714367 | 8.536080185 | 32 | 4 | 7 | 2 | Co_Occurence | c.2957delA, c.4284delT | 4/9 |
| c.9253delA | c.1561delT | 0.027037247 | 11.2396389 | 30 | 4 | 1 | 10 | Co_Occurence | c.1561delT, c.9253delA | 4/11 |
| c.3860delA | c.9800delA | 0.029684977 | 4.834815654 | 13 | 17 | 4 | 11 | Co_Occurence | c.3860delA, c.9800delA | 17/15 |
| c.3214delC | c.1016delA | 0.030654563 | 0.104395553 | 17 | 1 | 17 | 10 | Mutually_Exclusive | c.1016delA, c.3214delC | 1/27 |
| c.4284delT | c.1961delA | 0.030681174 | 9.493757478 | 26 | 5 | 13 | 1 | Co_Occurence | c.1961delA, c.4284delT | 5/14 |
| c.1961delA | c.3329delA | 0.030706115 | 4.570770686 | 19 | 12 | 8 | 6 | Co_Occurence | c.1961delA, c.3329delA | 12/14 |
| c.1561delT | c.7543delA | 0.032692209 | 9.644386826 | 35 | 3 | 5 | 2 | Co_Occurence | c.1561delT, c.7543delA | 3/7 |
| c.10248delA | c.3329delA | 0.033654314 | 9.786888944 | 24 | 6 | 14 | 1 | Co_Occurence | c.10248delA, c.3329delA | 6/15 |
| c.8053delA | c.3329delA | 0.033654314 | 9.786888944 | 24 | 6 | 14 | 1 | Co_Occurence | c.3329delA, c.8053delA | 6/15 |
| c.7667delA | c.5934delT | 0.033756563 | 6.689078777 | 32 | 4 | 6 | 3 | Co_Occurence | c.5934delT, c.7667delA | 4/9 |
| c.4169delT | c.3982delT | 0.033823925 | 5.746199577 | 29 | 5 | 8 | 3 | Co_Occurence | c.3982delT, c.4169delT | 5/11 |
| c.7177delA | c.3248delA | 0.035410421 | 7.301561568 | 31 | 4 | 2 | 8 | Co_Occurence | c.3248delA, c.7177delA | 4/10 |
| c.7177delA | c.4284delT | 0.035410421 | 7.301561568 | 31 | 4 | 2 | 8 | Co_Occurence | c.4284delT, c.7177delA | 4/10 |
| c.2588delA | c.9800delA | 0.038887941 | 8.787160016 | 23 | 6 | 15 | 1 | Co_Occurence | c.2588delA, c.9800delA | 6/16 |
| c.8021delA | c.9253delA | 0.039335967 | 4.396181686 | 24 | 8 | 6 | 7 | Co_Occurence | c.8021delA, c.9253delA | 8/13 |
| c.3248delA | c.10248delA | 0.039369876 | 8.088661764 | 35 | 3 | 4 | 3 | Co_Occurence | c.10248delA, c.3248delA | 3/7 |
| c.3248delA | c.7667delA | 0.039369876 | 8.088661764 | 35 | 3 | 4 | 3 | Co_Occurence | c.3248delA, c.7667delA | 3/7 |
| c.3248delA | c.8053delA | 0.039369876 | 8.088661764 | 35 | 3 | 4 | 3 | Co_Occurence | c.3248delA, c.8053delA | 3/7 |
| c.5297delA | c.3329delA | 0.040858533 | 4.708908207 | 22 | 8 | 12 | 3 | Co_Occurence | c.5297delA, c.3329delA | 8/15 |
| c.7177delA | c.1961delA | 0.040975951 | 4.429558567 | 23 | 8 | 10 | 4 | Co_Occurence | c.1961delA, c.7177delA | 8/14 |
| c.5073delA | c.1016delA | 0.044717709 | 5.980732947 | 25 | 6 | 12 | 2 | Co_Occurence | c.1016delA, c.5073delA | 6/14 |
| c.3982delT | c.1016delA | 0.045485431 | 0.188383209 | 16 | 2 | 16 | 11 | Mutually_Exclusive | c.1016delA, c.3982delT | 2/27 |
| c.5934delT | c.5297delA | 0.047661512 | 4.625064948 | 29 | 5 | 6 | 5 | Co_Occurence | c.5297delA, c.5934delT | 5/11 |
| c.3248delA | c.3982delT | 0.048807498 | 6.325098303 | 30 | 4 | 9 | 2 | Co_Occurence | c.3248delA, c.3982delT | 4/11 |
| c.5297delA | c.7667delA | 0.049580406 | 5.604591933 | 31 | 4 | 3 | 7 | Co_Occurence | c.5297delA, c.7667delA | 4/10 |
